# Comparison of CAD and Voxel-Based Modelling Methodologies for the Mechanical Simulation of Extrusion-Based 3D Printed Scaffolds

**DOI:** 10.3390/ma14195670

**Published:** 2021-09-29

**Authors:** Gisela Vega, Rubén Paz, Andrew Gleadall, Mario Monzón, María Elena Alemán-Domínguez

**Affiliations:** 1 Mechanical Engineering Department, Campus de Tafira Baja, Universidad de Las Palmas de Gran Canaria, 35017 Las Palmas, Spain; mario.monzon@ulpgc.es (M.M.); mariaelena.aleman@ulpgc.es (M.E.A.-D.); 2 Wolfson School of Mechanical and Manufacturing Engineering, Loughborough University, Loughborough LE11 3TU, UK; a.gleadall@lboro.ac.uk

**Keywords:** tissue engineering, scaffold, material extrusion additive manufacturing, 3D geometry modelling, finite element analysis, mechanical properties

## Abstract

Porous structures are of great importance in tissue engineering. Most scaffolds are 3D printed, but there is no single methodology to model these printed parts and to apply finite element analysis to estimate their mechanical behaviour. In this work, voxel-based and geometry-based modelling methodologies are defined and compared in terms of computational efficiency, dimensional accuracy, and mechanical behaviour prediction of printed parts. After comparing the volumes and dimensions of the models with the theoretical and experimental ones, they are more similar to the theoretical values because they do not take into account dimensional variations due to the printing temperature. This also affects the prediction of the mechanical behaviour, which is not accurate compared to reality, but it makes it possible to determine which geometry is stiffer. In terms of comparison of modelling methodologies, based on process efficiency, geometry-based modelling performs better for simple or larger parts, while voxel-based modelling is more advantageous for small and complex geometries.

## 1. Introduction

In tissue engineering applications, porous structures are desired for promoting cell growth and tissue regeneration. The morphology, size, and distribution of the pores have a vital effect not only on the mechanical properties of the structure [1], but also on its biological performance. Pore size has an optimal value for each type of tissue. Pore size below the optimal range hinders the vascularisation of the structure and cell migration. On the other hand, large pore sizes lead to a reduced surface area (and, therefore, a limited cell adhesion) and weak structures when biodegradable polymers are used to manufacture the structure. For instance, the osseointegration process is enhanced when scaffolds with pore sizes between 150 and 500 µm are used [2,3,4]. Moreover, the vascularisation achieved with interconnected pores with sufficient pore size enhances the osteogenesis process [5].

In addition to its role in the biological processes that take place during in vivo regeneration of the target tissue, the porosity is relevant during the degradation process of biodegradable scaffolds [6], as it is related to the permeability of the structure and, consequently, to the removal of the degradation by-products. These substances have an autocatalytic effect; hence, their local concentration has a strong impact on the degradation rate and mechanism of the scaffold [7]. 

Several techniques are used in tissue engineering for scaffold fabrication, which are the conventional ones (particulate leaching, phase separation, gas-foaming, or emulsion freeze-drying), as well as electrospinning and additive manufacturing (AM) techniques. In recent years, this last method has been the most selected one. AM allows a high degree of control of porosity, and this is one of the reasons for its popularity in the tissue engineering field [8,9,10,11]. 

AM techniques can be grouped into seven different categories according to ISO/ASTM 52900:2015: binder jetting, directed energy deposition, material extrusion, material jetting, powder bed fusion (e.g., selective laser sintering), sheet lamination, and vat photo-polymerisation (e.g., stereolithography). Among them, the most widely used technology for scaffold manufacturing is material extrusion (MEX), commonly known as extrusion-based 3D printing or fused deposition modelling (FDM). FDM presents ease and flexibility in the processing and selection of materials, as well as in the manufacturing process [8,9,10,11,12,13,14].

However, in the case of material extrusion AM, scaffolds are not always designed in the same way. Some authors define the solid part and then set its porosity in the printing settings of the slicer software [15,16,17]. In this step, the 3D printing path is determined according to the printing settings, and the resulting geometry is shown in the software. Nevertheless, this geometrical information cannot be exported and, therefore, it cannot be used for the prediction of mechanical behaviour through simulation techniques such as finite element analysis (FEA).

Other researchers preferred firstly to design the model using CAD software, representing it with filaments instead of as a solid part, and then print the scaffold [10,14,18,19,20,21,22,23]. In those cases, they were able to simulate the model behaviour and compare the results with the experimental ones or optimise the part before printing it. Despite this, the printing process of this method is not suitably defined; it could present problems depending on the slicer software chosen, as each filament is typically interpreted as a solid piece and represented as several filaments, instead of a single pass.

On the other hand, there are several valid options to simulate the mechanical performance of the printed scaffold based on its model, such as the homogenisation [24,25,26,27], the voxel-based [12,28,29], or the CAD-based modelling techniques [10,14,18,19,20,21,22,23,30,31].

The CAD-based modelling method is the most widely used. There are several ways to obtain the geometric model. One of them is the definition of a layer, which is repeated along the height, combining the direction of the filaments. This method has the limitation that it is only applicable to quadrangular prisms or cylinders with the same mesostructured layers (when the base figure is rotated 90°, it keeps its shape). Another way is to model the deposited part by computer-aided software based on the G-code information. This method can reproduce simple geometric objects that are defined by their boundaries (vertices, edges, and loops). However, it could raise problems if it is applied to non-Eulerian solids or biomorphic porous structures. Moreover, it could also fail if there are gaps or overlaps, and its manipulation may be difficult when objects have fine internal architectures or are too large [32,33].

To solve the computational limitations of CAD models, unit cells are used. These are basic building blocks that represent the scaffold microarchitectures. There are unit cell libraries available, which, combined with the homogenisation technique (application of the unit cell properties to the entire solid), make the computational method more efficient [32,33].

On the other side of modelling methods, the image-based approach is also used and is compatible with computed tomography (CT) and magnetic resonance imaging (MRI). It allows the internal architecture to be controlled by the intersection between 3D binary images which define the voxel values (solid or void). However, this method presents the limitations of resolution and dataset dimensions and the need for a reference image [32,33].

Recent studies have introduced another way of modelling porous structures using natural geometries as reference (e.g., bones). This modelling technique takes into account the irregularities and the spatial evolution of the reference porous structure. This is the top-down design through the Voronoi tessellation method. It considers the irregularity of bones and controls the distribution and shape of the pores, in addition to the gradient interconnected porosity. The main challenge of this method is to find a suitable manufacturing technology, which seems to be the selective laser melting (SLM) one [34,35].

The development of a modelling method combined with a simulation tool to analyse the mechanical behaviour of different pore sizes and structures would allow reducing the experimental work needed when developing a new design for a scaffold to be used in tissue engineering. Therefore, this tool would improve the cost-efficiency of the development process. 

This is the first study to evaluate and compare several modelling techniques, particularly geometry-based and voxel-based ones. This work developed a new automated modelling technique, which is the first to define the methodology for modelling any CAD part from the G-code file, from simple parts to more complex ones. Furthermore, the new technique was evaluated in comparison to the only other modelling approach that is able to simulate microscale geometry in practical-sized structures, the VOLCO software [28]. On the other hand, a methodology is defined to make use of the models obtained to estimate the mechanical behaviour of the parts before printing them, which would allow their optimisation. As a case study, FEA was applied to 3D printed scaffolds (material extrusion) in order to compare their capabilities and limitations in the modelling and simulation process. Several scaffolds were designed by defining different porosities and infill patterns, and the modelling and FEA simulation were carried out to compare the results between both methodologies. Moreover, some samples were also manufactured and tested to compare the simulations with the experimental results.

## 2. Materials and Methods

### 2.1. Materials

The printing material employed for manufacturing the scaffolds used in this experiment was polycaprolactone (PCL), a biodegradable polyester with suitable properties for tissue engineering applications. The material properties are shown in Table 1.

### 2.2. Geometries and Manufacturing Parameters

A solid, cylindrical scaffold (Ø10 × 7 mm) was used for this study. This solid part was introduced in the slicer software (Slic3r 1.3.0) (Alessandro Ranellucci, Rome, Italy), and three different printing settings were selected to obtain three parts with different fill patterns (rectilinear and gyroid) and fill densities (40% and 50% density, corresponding to 60% and 50% porosity, respectively). Table 2 shows the printing parameters of the three configurations studied: a scaffold defined by a rectilinear pattern and a porosity of 50% (50_rect), a scaffold similar to the first one but with a porosity of 60% (60_rect), and another one with a gyroid fill pattern and a porosity of 50% (50_gyr).

After setting the scaffold characteristics, the G-code file for printing and modelling was obtained with Slic3r 1.3.0.

### 2.3. Modelling Methods

To be able to apply FEA to the final printed part, a modelling strategy is needed to reproduce the deposited part. This work compares two different approaches to obtain the 3D CAD part from the G-code file: voxel-based and geometry-based modelling techniques.

#### 2.3.1. G-Code Conditioning

A Matlab 2021a script processes the G-code and conditions it to be used in whichever of the modelling techniques. The first step is the conversion of the G-code into a coordinate file. This is a spreadsheet (.xls) which consists of four columns that contain all the movement coordinates and deposition guidelines. From the first to the third column, the file contains the *x*-, *y*-, and *z*-coordinates, respectively, of each point of the path. The fourth column indicates, in a binary format, if that point is the first one of the movement (0) or an intermediate or final point of the path (1).

The coordinates are extracted from the G-code reading it and looking for its specific denomination, *X*, *Y*, or *Z*, followed by a number. In the spreadsheet, only the corresponding number is collected.

To fill the fourth column, comments are required in the G-code; thus, the verbose option must be selected during the G-code generation. Some key comments for starting a new path are detected to set a “0” in the first point of that path. This step is automatically done by the Matlab script that obtains the coordinates from the G-code file. This script was configured to be able to manage different slicer software, which uses different comments to indicate the positioning movements. Some examples of these movements are “move to first infill point” or “move to next layer”, specific to the file obtained in Slic3r 1.3.0 or, in the case of Simplify 3D 4.1.1., “layer <number>”, “feature infill”, “feature outer perimeter”, or “feature solid layer”.

#### 2.3.2. Voxel-Based Modelling (VOLCO)

**Figure 1:**
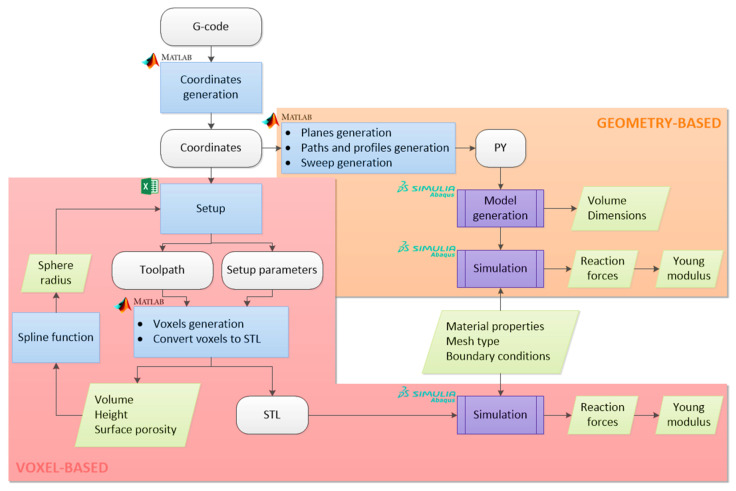
Flowchart of the modelling methodologies.

In the voxel-based methodology, the tool developed at the University of Nottingham, Volume Conserving Model for 3D Printing (VOLCO) (run in Matlab R2021a), was used [28]. It is executed by macros of a datasheet file, called “Setup.xls”, which needs the introduction of the coordinates of the part. These coordinates can be obtained from a G-code file with the Matlab script described above. The scheme of the voxel-based modelling process from the G-code to the results of the mechanical behaviour is shown in Figure 1.

VOLCO simulates the material extrusion during the manufacturing process and generates a voxelised 3D-geometry model of the predicted microarchitecture. The software deposits voxelised spheres, placed one after the other, to simulate the filaments, and, when they interact, there is an expansion of the material in the available space, keeping the volume constant. This predictive model starts from the premise that the deposited filaments are not perfect cylinders, but that the molten material interacts with those obstacles that are encountered in the deposition process [28].

Once VOLCO has been executed, a voxelised model is obtained and represented in an STL file. Additionally, the dimensional and porosity data are also depicted, i.e., the volume, height, and porosity of the part. The STL can be imported into Abaqus/CAE 6.14-1 (Dassault Systèmes Simulia Corp., Providence, RI, USA) and, after the FEA, the reaction forces can be determined and, therefore, the Young’s modulus of the part can be deduced.

The procedure previously described is the basic one, but an additional step can be applied to improve the accuracy of the resulting voxelised volume. This consists of an iterative process to equalise the volume of the voxelised part and the theoretical one, according to the G-code. It is done through the spline function of Matlab R2021a (The Mathworks Inc., Natick, MA, USA). In an iterative process, this function is manually applied with the available data (sphere radius applied and volume of the model obtained) to estimate, by cubic spline interpolation, the correct sphere radius in the VOLCO setup (“OriginalSphereRadius” in the Setup.xls) that will lead to a voxelised model with the theoretical volume of the G-code.

#### 2.3.3. Geometry-Based Modelling

The second method is the automated sweep CAD modeller (run in MATLAB R2021a) of the extrusion-based G-code (DECODE). This consists of a Matlab script that reads the previously mentioned coordinates file. This information is used to automatically write a Python 3.9.6 (Python Software Foundation, Beaverton, OR, USA) file with the code that generates the planes that represent each layer, the sketches of the filament paths and profiles (sections), and the sweep operations to model the deposited part in Abaqus/CAE 6.14-1. This Python file can be subsequently run in the FEA software (Abaqus/CAE 6.14-1) to obtain and visualise the CAD model.

**Figure 2:**
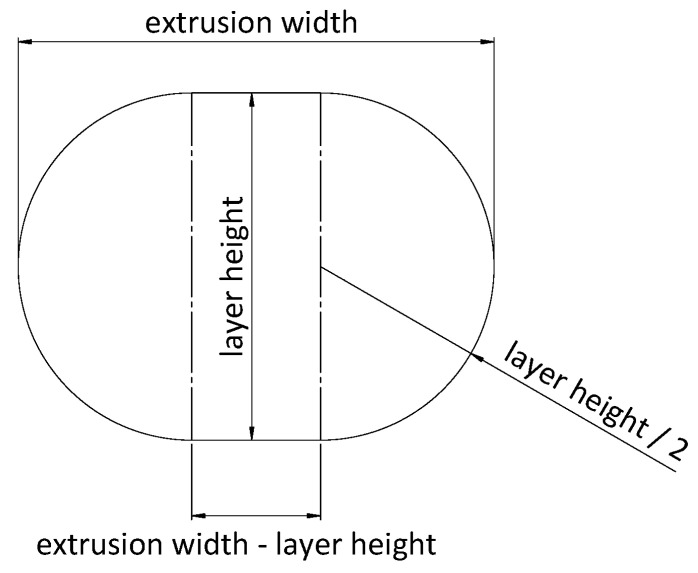
Filament profile shape and dimension.

As mentioned above, a plane is defined at each printed layer height, where the filament is deposited. To sweep each layer, it is necessary to have drawn the sketches of the path and the profiles (section of the deposited filament). The sketches of the profiles are positioned at the beginning of the path, perpendicular to the first line direction. The shape of the profile is the combination of a rectangle and two semicircles, as represented in Figure 2.

Several authors have defined how the profile of a printed filament should be represented. Some of them affirmed that the cross-section must be an ellipse, such as Bellehumeur or Rupal [30,36]. However, the solution presented in this work is more similar to the elliptic–rectangular cross-section proposed by Park and Rosen [37] and supported by Gleadall [28]. This shape was assumed by taking into account the deformation of the fused filament when it is deposed on a plane layer and the effect of the extruder nozzle running through the top layer.

After running the Python file, the model is obtained in Abaqus/CAE 6.14-1. After these steps, the volume and dimensions of the part can be obtained. The model is then prepared for the simulation, taking into account the material properties, mesh type, and boundary conditions. Once all the steps are completed, the mechanical behaviour such as displacements or reaction forces can be measured. The scheme of the complete process is shown in Figure 1

During the development of the geometry-based modelling technique, some CAD modelling or meshing issues were observed. For these reasons, several strategies were developed and tested to obtain a model without problems in Abaqus/CAE 6.14-1. These strategies were “whole-part modelling”, “line-by-line modelling”, “line-by-line modelling with corner revolutions”, and “modelling by sections”. 

To test the effectiveness of these strategies, a simple part, test specimen (40 × 5 × 2 mm^3^), and a complex part, the gyroid scaffold, were modelled.

1. Whole-Part Modelling

The first and simpler methodology was “whole-part modelling”. In this strategy, each path corresponds to a continuous deposition made by the extruder. In case that there is a discontinuity in the path of the same layer, each part of the path is represented through a separate sweep feature. The sketches of the paths are represented by straight lines that consecutively join each point of the coordinates file between 0 and 1 in the fourth column, which correspond to all the consecutive coordinates of continuous deposition. These coordinates are located in all the direction changes of the printhead during the deposition.

**Figure 3:**
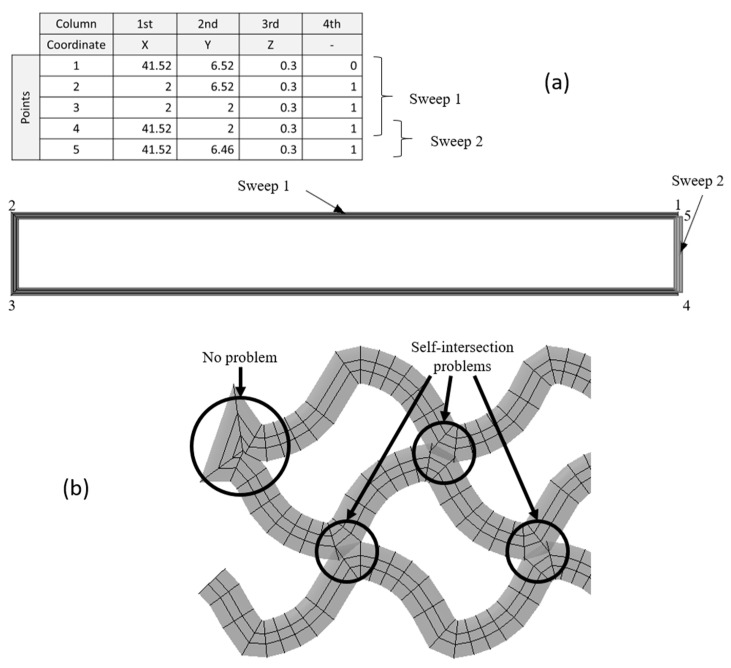
Division of close sweep (**a**) and self-intersection of a gyroid fill pattern (**b**).

This type of model presents sharp corners in very acute angles, and, in the case of a close sweep, Abaqus/CAE 6.14-1 gives an error in the modelling process. This last problem can be solved by automatically checking this condition (automated modelling). If the first and the last points of a sweep are too close (distance < extrusion_width/2), it is considered a close sweep, and, as a consequence, a division is applied by separating the last line of the sweep into a new sweep, as shown in Figure 3. Otherwise (open path), the sweep does not need to be divided.

Although this additional checking solved the modelling error, when this modelling strategy is applied to more complex parts such as a scaffold with a gyroid fill pattern, the model presents self-intersections that often result in meshing problems in Abaqus/CAE 6.14-1 (overlapped volumes). An example of a self-intersection is shown in Figure 3.

2. Line-by-Line Modelling

**Figure 4:**
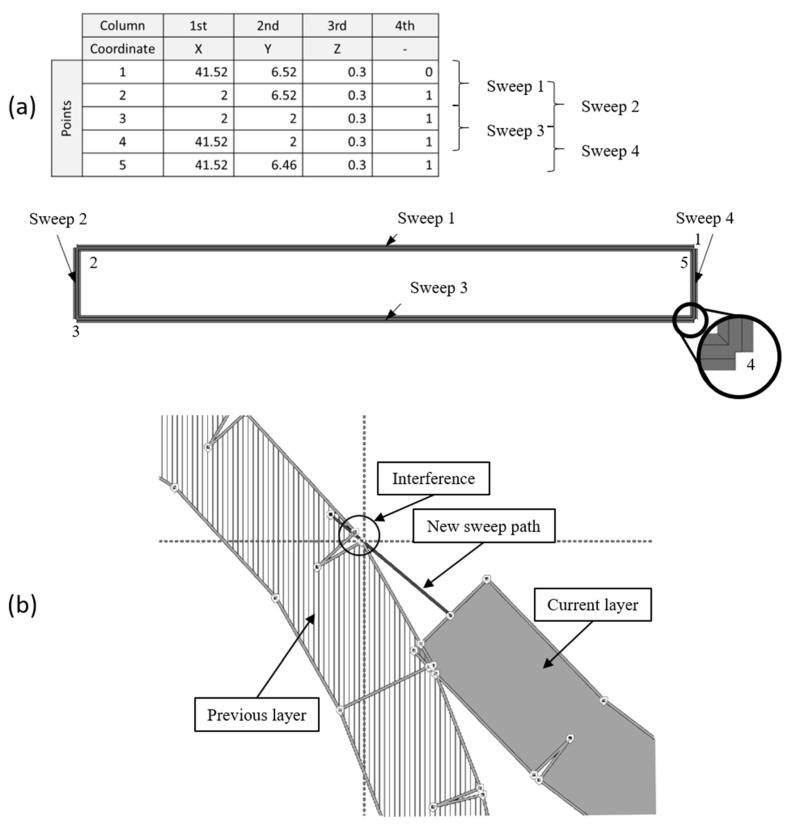
Line-by-line modelling (**a**) and its error (**b**).

To avoid the problems of “whole-part modelling”, another strategy called “line-by-line modelling” was developed, which consists of creating a sweep per line, thus resulting in empty corners at the joints (Figure 4).

This new strategy led to new problems previously undetected in Abaqus/CAE 6.14-1. The error in the modelling process appears while trying to sweep certain lines of the path that interfere with points present in previous layers, corresponding to the corners created in the empty joints. The error is shown in Figure 4.

3. Line-by-Line Modelling with Corner Revolutions

**Figure 5:**
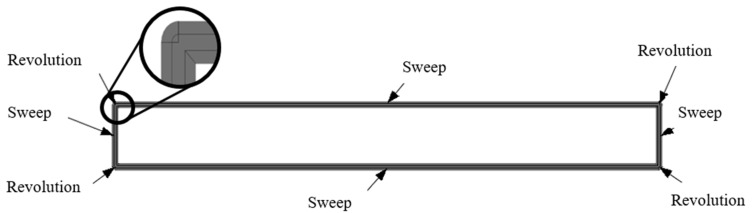
Line-by-line modelling with corner revolutions.

The previously mentioned error was solved by filling the joints with revolutions. This led to a new strategy called “line-by-line modelling with corner revolutions”. The result of this technique is shown in Figure 5. This strategy requires the creation of planes perpendicular to the path at each joint to create the corresponding sketches (the same filament profile depicted in Figure 4) for the revolution feature with respect to the vertical symmetric axis. As a consequence, each line and corner would require several operations, which would slow down the modelling process. For this reason, it was decided to find another strategy that does not require the separation of each line from the path but avoids self-intersections, which is the “modelling by sections”.

4. Modelling by Sections

The last strategy proven was the “modelling by sections”, where the sweeps that present self-intersection are divided to avoid the previous error. Although the model presents sharp corners as in the “whole-part modelling” strategy, it was later proven that their influence is not too relevant from the mechanical point of view, at least for the prediction of the stiffness in static conditions.

**Figure 6:**
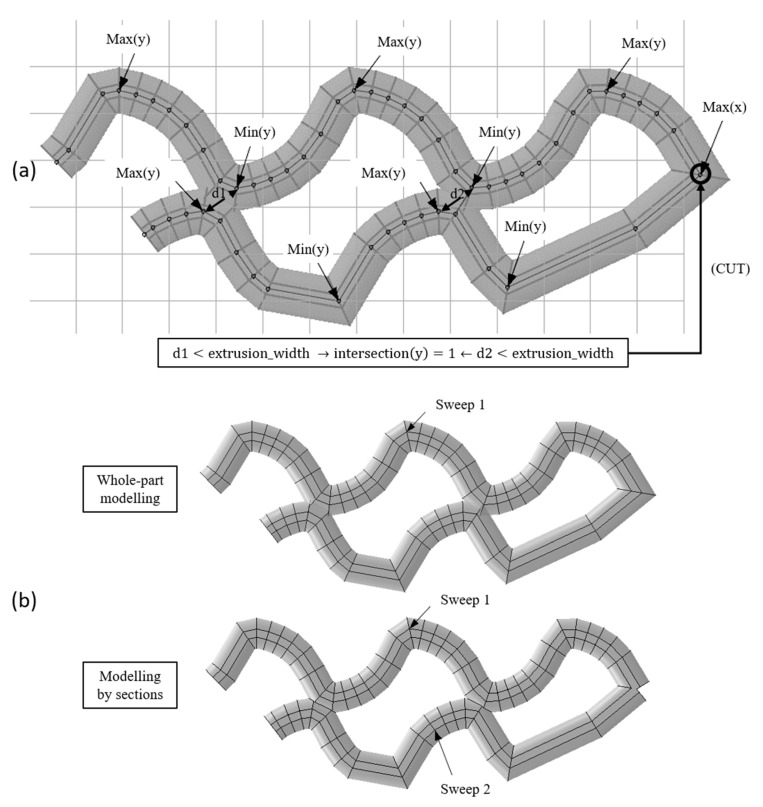
Self-intersection identification and division process (**a**) and its result (**b**).

The “modelling by sections” strategy identifies the peaks of each path (relative maximums and minimums) in *x-* and *y*-coordinates. Then, it compares the distance between all the relative maximums and minimums of the *x*-coordinate on the one hand and, on the other hand, all the relative maximums and minimums of the *y*-coordinate. If there is any distance lower than the extrusion width and there are at least three points between the two to be compared, there is a self-intersection in that coordinate (*x* or *y*). The reason for excluding points that are located close is that, in those cases, there would be no self-intersection in modelling, since the self-intersection problem only arises when the path separates from the previous path and intersects again (Figure 6).

If there is a self-intersection in *x*-coordinate, the path is divided into the *y*-coordinate peaks, and vice versa. This cut is represented in the coordinate matrix as “−1” in the fourth column. It works differently than “0” as it is the last point of the previous sweep and the first point of the next one.

An example of a division process is shown in Figure 6, in which the distances between peaks are higher than the extrusion width, except for d1 and d2. These self-intersections are located at the peaks of the *y*-coordinate; therefore, a cut at the *x*-coordinate peak is assigned “−1” in the fourth column.

However, there is also the possibility that there is a self-intersection in the sweep, but that it does not exactly coincide with the peaks. Therefore, if two points are near (the distance is greater than the extrusion width but smaller than the 150% of this width), it also compares the maximum and its two previous and following points with the minimum and its two previous and following points. 

**Figure 7:**
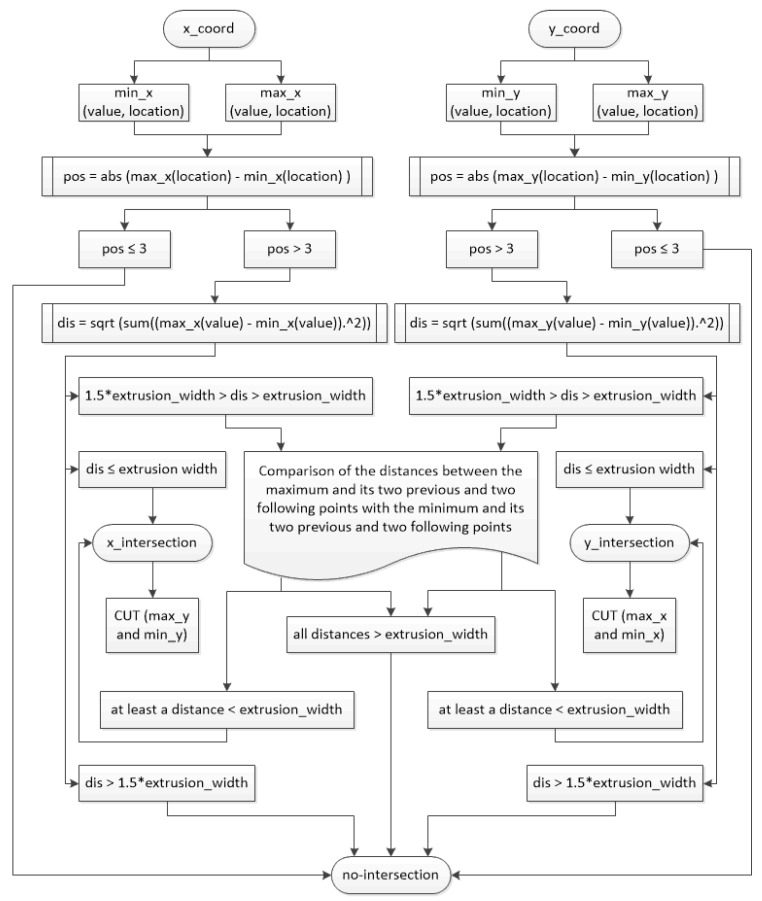
Modelling by sections diagram.

The diagram of the whole process and conditions for section divisions is shown in Figure 7.

**Figure 8:**
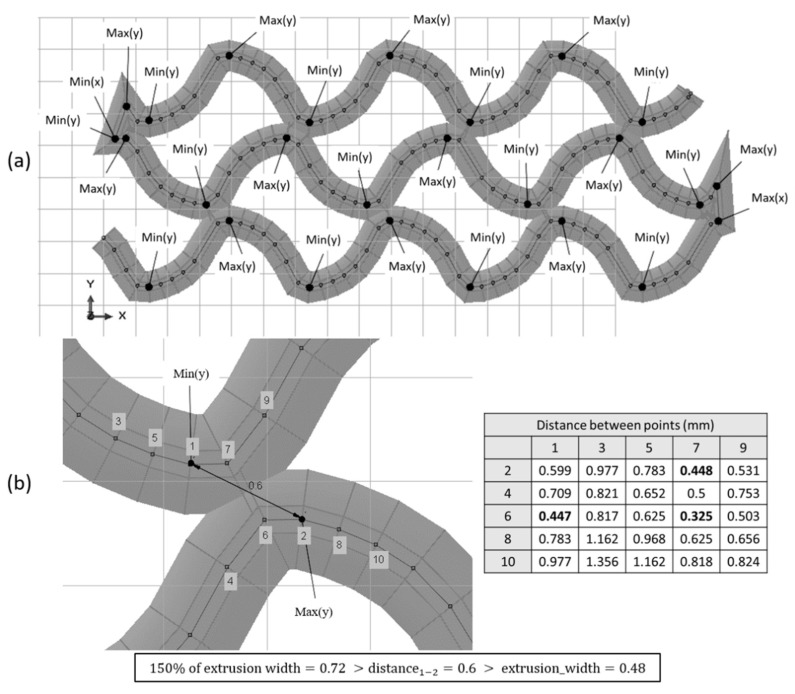
Peak identification in a complex geometry (**a**) and distance comparison of peaks and their previous and following points (**b**).

As another example, in Figure 8, the identification of relative maximums and minimums of a sweep path is shown. Some of them fulfil the condition of being at a distance smaller than the extrusion width. However, an example of a maximum and a minimum that are close is shown, but the points that make the sweep auto-intersect are not the peaks at points 2 and 7, 1 and 6, or 6 and 7.

**Figure 9:**
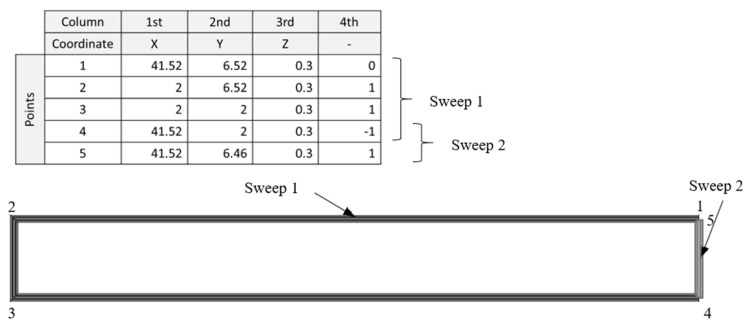
Close sweep in modelling by sections.

The “−1” value is also used to indicate the cut of a close sweep, applying the same process as in the “whole-part modelling” to detect it, as shown in Figure 9. It should be noted that, if there are no self-intersections in the model, the resulting model will be the same as that obtained with “whole-part modelling”. Thus, this last modelling strategy is suitable for complex geometries, while “whole-part modelling” is useful only for simple parts.

5. Comparison of Modelling Strategies

**Figure 10:**
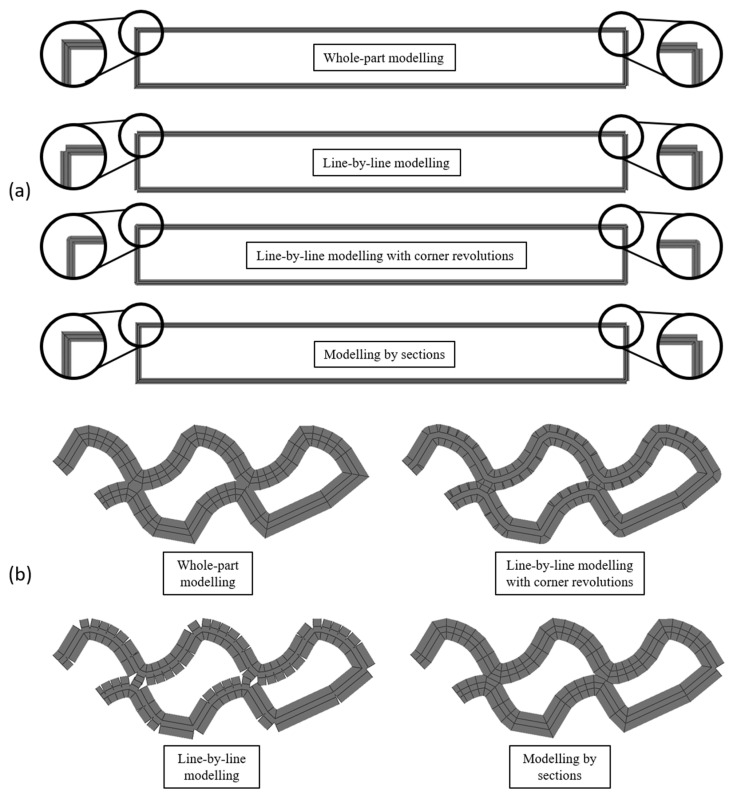
Comparison of modelling strategies in simple parts (**a**) and complex parts (**b**).

The resulting shape differences between the developed modelling strategies are shown in Figure 10 for simple and complex parts (with self-intersection). Empty corners are found in the division of the last section of “whole-part modelling” and “modelling by sections”, in the separation of self-intersecting paths in “modelling by sections”, and between each section of “line-by-line modelling”. Sharp corners can be found in both “whole-part modelling” and “modelling by sections”. Lastly, it can be observed that, in complex parts, “line-by-line modelling”, “line-by-line modelling with corner revolutions”, and “modelling by sections” avoid self-intersections.

To validate the modelling strategies, the test specimen previously defined was modelled following the DECODE methodology steps. To obtain the G-code file, the solid part was introduced in Slic3r 1.3.0, and the printing parameters listed in Table 3 were set.

**Figure 11:**
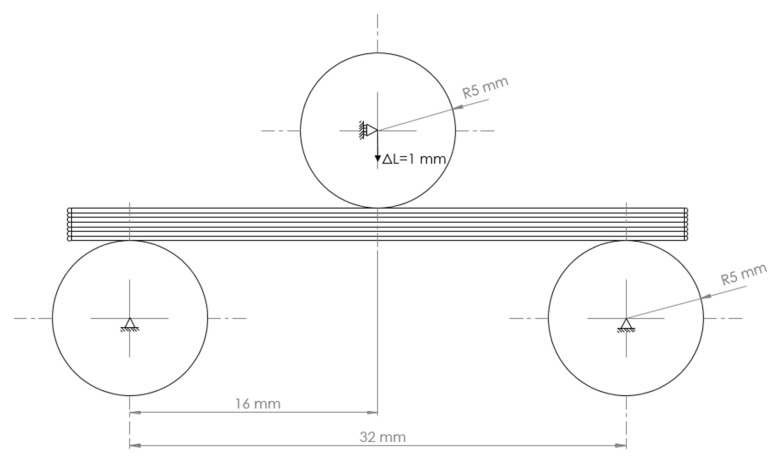
Flexural test of test specimen.

The modelling strategies used were “whole-part modelling”, “line-by-line modelling”, “line-by-line modelling with corner revolutions”, and “modelling by sections”. All the models obtained were simulated with a three-point flexural test in Abaqus/CAE 6.14-1, as shown in Figure 11. For this purpose, three rigid cylindrical parts with a radius and a height of 5 mm were designed, and surface-to-surface interaction was defined between the test specimen and each cylinder. A friction coefficient of 0.15 was assigned to the tangential behaviour of the interaction, and the normal behaviour was defined as a hard contact. The models were meshed with a second-order tetrahedral mesh and a seed size of 1 mm. The supports were encastred, and a downward vertical displacement of 1 mm was applied to the crosshead. After the definition of all the above conditions, the reaction forces obtained in the different cases were as shown in Table 4.

The resultant reaction forces did not vary significantly among the different modelling strategies, and it was found that they were the same in “whole-part modelling” and “modelling by sections”, since, in parts without self-intersection, their structures were the same. For this reason, it was decided to choose “modelling by sections” as the best strategy, since fewer modelling errors arose, and the modelling strategy is relatively easy to automate.

#### 2.3.4. Compression Simulation by Finite Element Analysis

In order to obtain the stiffness and elastic modulus of the scaffolds models, they were simulated in Abaqus/CAE 6.14-1 by FEA. In particular, a compression test was applied with the following boundary conditions: the vertical movement was restricted at the base of the scaffold, two points of this base were encastred to avoid displacement in the horizontal plane, and a vertical displacement of 0.2 mm was applied on the top layer to simulate the compression movement of the compression plate. 

Another important part of the simulation conditioning is the material properties. The selection of the model that defines the material behaviour may have a great influence on the results. In this case, linear behaviour of the elastic material (isotropic) was assumed (based on previous mechanical tests) and nonlinear effects of large deformations and displacement were included. Considering the selected material behaviour, the density and tensile elastic modulus were introduced according to the datasheet of the PCL, presented in Table 1, and the Poisson’s ratio was defined as 0.46, according to some studies [38,39]. Although several physical parameters of the material may have an effect on the mechanical behaviour, the most relevant properties are the elastic modulus and Poisson’s ratio. However, other models could be applied depending on the availability in the FEA software (hyperelasticity, plasticity, nonlinear behaviour, anisotropy, etc.). Each of these models would require different physical parameters.

On the other hand, regarding the mesh type definition, geometry-based models were meshed with C3D10 (10-node quadratic tetrahedron) and C3D4 (four-node linear tetrahedron) element types, and the voxel-based models were meshed only with C3D4, the default element type for converting voxels to tetrahedrons. This difference was due to the fact that the voxelised model does not allow meshing with second-order elements. The seed size of the mesh was varied depending on the part in order to avoid distorted elements, as well as the application of virtual topology. The meshing conditions for each model are shown in Table 5. Moreover, rigid joints between layers (a single solid) were assumed.

During the model conditioning step in Abaqus/CAE 6.14-1, it was not possible to mesh the CAD model of the 50_gyr scaffold with quadratic elements.

After the simulation, the reaction forces were obtained in the base layer and, therefore, the Young’s modulus was evaluated according to Equation (1), extracted from ISO 604:2002 (plastics—determination of compressive properties). Note that the equivalent area corresponds to the area of the base as if it were a solid part.

(1) $$E=\frac{\sigma }{\varepsilon }=\frac{F/A}{∆L/L_{0}}=\frac{F\cdot L_{0}}{A\cdot ∆L},$$

where E is the Young’s modulus or modulus of elasticity (MPa), σ is the tensile stress (MPa), ε is the deformation, F is the reaction force (N), A is the equivalent area (mm^2^), ∆L is the displacement (mm), and L_0_ is the original height (mm).

### 2.4. Scaffold Manufacturing and Compression Tests

The real scaffolds were manufactured by FDM in a BQ Hephestos 2 3D printer. The extrusion temperature was set to 190 °C.

Four replicas per group were subjected to mechanical characterisation under compression load. The mechanical testing was carried out in a LIYI (LI-1065, Dongguan Liyi Environmental Technology Co., Ltd., Dongguan, China) testing machine in displacement control mode. The crosshead speed was set to 1 mm/min, and the compression modulus was calculated as the slope of the initial segment in the stress–strain graph.

### 2.5. Morphological Characterisation

Printed scaffolds were geometrically compared with models by microscope imaging to determine which representation was closer to reality. Concretely, the Olympus BX51 microscope (Olympus Co., Ltd., Tokyo, Japan) with a 2× magnification factor was used to collect images of the top of the three types of scaffolds (50_rect, 60_rect, and 50_gyr).

## 3. Results and Discussion

### 3.1. Modelling Efficiency

#### 3.1.1. Scaffold Modelling Efficiency

The modelling efficiency was estimated from the time and CPU required for modelling and simulation processes. The times required to generate the coordinates and to follow the modelling and simulation steps in each scaffold configuration and both modelling techniques are collected in Table 6, as well as the minimum memory required for simulation.

The computer used for modelling and simulation was an Intel^®^ Core™ i9-9820X CPU @3.30GHz, with 64.0 GB installed RAM and a 64-bit operating system, x64-based processor.

The use of C3D4 meshes saved memory in the simulation of the models, but not always time. In general, modelling and simulations using DECODE were more efficient, although they were very similar to VOLCO in complex geometries such as the gyroid scaffold, which also presented the difficulty of not being able to be meshed with second-order elements with the available software and hardware.

**Figure 12:**
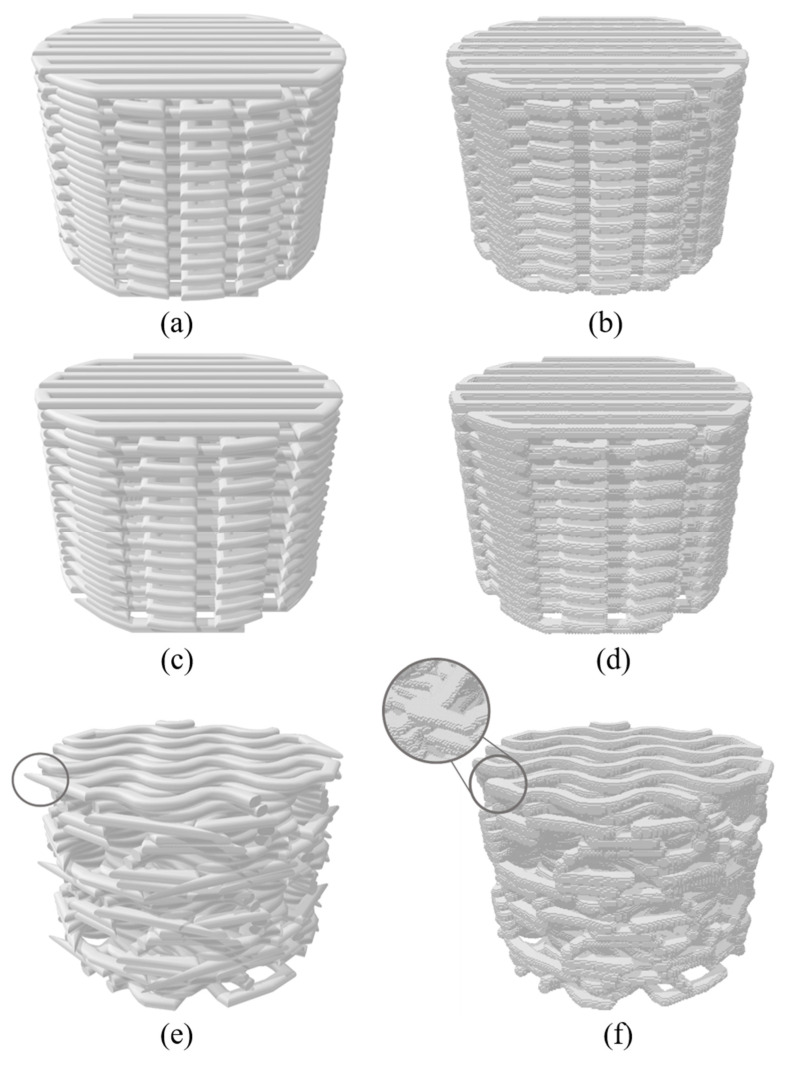
Models: (**a**) 50_rect DECODE (**b**) 50_rect VOLCO, (**c**) 60_rect DECODE, (**d**) 60_rect VOLCO, (**e**) 50_gyr DECODE and (**f**) and 50_gyr VOLCO.

The models generated through the different modelling techniques are presented in Figure 12.

#### 3.1.2. Modelling Limitations

Voxel-based and geometry-based modelling and simulation methodologies were studied in terms of applicability to the full range of FDM structures. In the particular case of dimensional and shape adequacy, limitations were found depending on the software and hardware used, since, for large or complex parts, Abaqus/CAE 6.14-1 cannot support simulation, meshing, or modelling. For example, for the large part presented in Figure S12, modelling was not possible from the python file. On the other hand, the 50_gyr scaffold and the corner part shown in Figure S13 were modelled but not meshed by Abaqus/CAE 6.14-1 due to complex geometry and large memory required to mesh the part, respectively. Lastly, in other cases, parts could be modelled and meshed but not simulated because the minimum memory required for FEA was higher than that available.

On the other hand, in both modelling techniques, Microsoft Excel was used to record the coordinates obtained from the G-code. In the most favourable case, using Microsoft Excel 64 bit, the worksheet was limited to 1,048,576 rows by 16,384 columns, but not to the maximum use of 2 GB of RAM as in the 32 bit version. In short, as each coordinate is saved in a new row, there would be a limit of 1,048,576 coordinates in VOLCO. In DECODE, this would not be a limitation as it allows the reading of several worksheets continuously, as well as in the coordinate matrix generated in Matlab R2021a, which would only present memory limitations due to the available hardware.

The computer used for modelling had 64.0 GB of installed RAM, but the memory available for all arrays was 43.7 GB. In addition, each element of the matrix required approximately 8 bytes of memory. Thus, the number of elements was limited to 5.86 × 10^9^. 

On the one hand, the array limitations and the four columns needed to define a coordinate set of 1.465 × 10^9^ as the maximum number of coordinates in the matrix.

On the other hand, this affected VOLCO in the construction of the voxel matrix, which would need as many elements as voxels (defined in a binary way) needed to define the part. This number depends on the dimensions and voxel size, as defined in Equation (2).

(2) $$\mathrm{No}.\;\mathrm{voxels}=\frac{\left(X_{\mathrm{end}}-X_{\mathrm{start}}\right)\cdot \left(Y_{\mathrm{end}}-Y_{\mathrm{start}}\right)\cdot \left(Z_{\mathrm{end}}-Z_{\mathrm{start}}\right)}{{\mathrm{VoxelSize}}^{3}},$$

where X_end_, Y_end_, Z_end_ are the maximum coordinates (mm), X_start_, Y_start_, Z_start_ are the minimum coordinates (mm), and VoxelSize is the set voxel size (mm).

Several examples are shown in Table 7 to determine the practical-sized structures that can be modelled by VOLCO. Those that require more memory than available cannot be modelled. It should be noted that a maximum voxel size of 100 μm was set to allow proper filament resolution, as the nozzle tip used was 0.4 mm, which allowed a maximum layer height of 0.3 mm.

In summary, Table 8 presents the steps that are possible or not for each part, using each modelling methodology, with the available hardware and software.

In all cases, the limitation for modelling, meshing, or FEA was the available memory, except for the meshing of the geometry-based model of the gyroid scaffold and the STL file generation of the large part by VOLCO. In the case of the scaffold, this was due to the limitations for meshing complex parts in Abaqus/CAE 6.14-1. In the case of VOLCO, the large part had 7,196,287 coordinates that could not be recorded in a single Microsoft Excel worksheet.

### 3.2. Dimensional Accuracy

#### 3.2.1. Volume

One of the most important parameters to compare the geometrical adjustment is the volume. It can be measured in the models through Abaqus/CAE 6.14-1 query tools, but VOLCO also generates an output with this information. The theoretical volume was extracted from the G-code file, which specifies the amount of filament used.

All the data collected are compared in Table 9. The deviation between the theoretical and the VOLCO model volumes was almost 0% because of the application of the spline function previously mentioned.

Compared with the theoretical data, the differences in volume were negligible. On the other hand, taking into account the deviations from the experimental results, between 1.29% and 3.11%, it could be assumed that the dimensional accuracy of the models is acceptable. This last statement is reinforced by the premise that the selected printing parameters and the temperature have a considerable influence on the final dimensions of the printed part, which do not always respect the theoretical measurements [40]. 

#### 3.2.2. Dimensions and Shape

Another important comparison involves the dimensions of the models compared with the initial solid design (theoretical) and the printed scaffolds (real), as shown in Table 10.

The differences between the theoretical scaffold and the models lie in the modelling techniques limitations, as shown in Table 8. In the case of the DECODE modelling, the sweep operation creates sharp tips in very acute angles. On the other hand, VOLCO modelling expands the filament in joints. However, another determining factor in the dimensional inaccuracy of FDM printed parts is nonuniform temperature gradients [41]. Furthermore, there are physicochemical characteristics of the materials such as glass transition temperature or free volume that cannot be considered in the modelling techniques, as both methods (geometry-based and voxel-based) utilise the G-code file to generate the geometry. This limitation could be relevant in other technologies such as electrospinning or rotary jet spinning [42]; however, in the case of material extrusion, the effect could be negligible.

**Figure 13:**
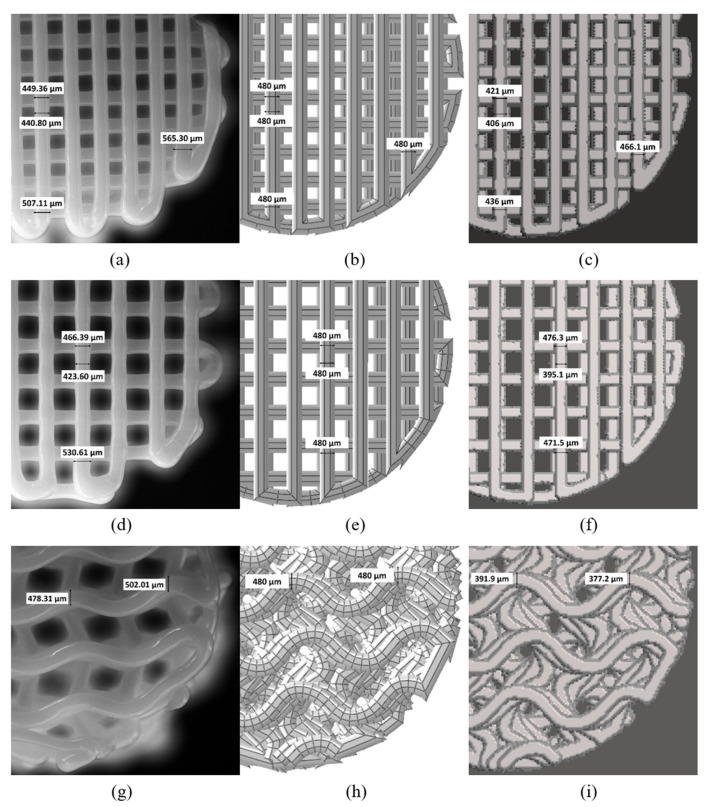
Filament width comparison of: (**a**) 50_rect microscopy image of printed scaffold, (**b**) 50_rect CAD model, (**c**) 50_rect voxelised model, (**d**) 60_rect microscopy image of printed scaffold, (**e**) 60_rect CAD model, (**f**) 60_rect voxelised model, (**g**) 50_gyr microscopy image of printed scaffold, (**h**) 50_gyr CAD model and (**i**) 50_gyr voxelised model.

The comparison of filaments shape and width between the models and printed scaffolds was done for 50_rect, 60_rect, and 50_gyr types, as shown in Figure 13. The images and measurements of the printed scaffolds were obtained by microscopy imaging. As is shown in Figure 13, the voxelised filaments did not have constant width as in reality, in contrast with the CAD model. However, the voxelised filaments had smaller widths than the other scaffolds. 

Therefore, the DECODE model could be an approximation of the printed part, representing filaments with non-variable width but with an average value similar to that along the path. On the other hand, the VOLCO model presented a final shape closer to the real scaffold, although the value of the filament width did not necessarily coincide.

### 3.3. Equivalent Elastic Modulus

The models for the different scaffold configurations were simulated with Simulia Abaqus/CAE 6.14-1. A compression test was applied to each model; accordingly, the reaction forces, which appear in the base layer, and the Young’s moduli were obtained. The results of the simulations and the experimental tests are collected in Table 11.

During the simulation step, it was not possible to mesh the CAD model of the 50_gyr scaffold, but it was possible to obtain a meshed 50_gyr model with linear elements, trying multiple seed sizes until finding that which did not generate distorted elements. The meshing problem is one of the most recurrent issues in geometry-based modelling methodology, especially in complex geometries. In these cases, voxel-based modelling is more suitable.

The comparison of the results of both modelling techniques shows that the voxel-based model was stiffer than the geometry-based one. This is because VOLCO modelling takes into account the intersection of the filaments, expanding the material at the joints. This leads to larger sections of material in the contact between layers, consequently increasing the stiffness of the part under compression compared to DECODE modelling.

The mesh type used also influenced the stiffness of the part, as can be seen in the different geometry-based models. Linear elements (C3D4) were less precise and, therefore, tended to increase the stiffness of the model.

Another conclusion that can be extracted from the elastic modulus results is that related to the prediction of the part stiffness. As previously demonstrated and presented in Table 11, there is a large dimensional deviation between the printed parts and the theoretical values. Moreover, during the deposition process, voids may appear, leading to a higher porosity and consequently, affecting the mechanical properties. This effect depends on the materials used, especially in composite materials [43]. Therefore, both the dimensional deviation and the voids arising lead to differences between the simulated and the experimental mechanical results. Using the current procedure, it is not possible to predict the exact value of the Young’s modulus, whether with the VOLCO or DECODE modelling techniques, but it is possible to accurately compare different geometries. In other words, the modulus variations are not proportional between the experimental and simulation results, but the most rigid model according to the simulations corresponds with the most rigid part.

## 4. Conclusions

This is the first study to consider modelling the printed parts before manufacturing to apply FEA and to compare which modelling technique is more suitable, practical, and efficient for a specific case. Furthermore, a new CAD-based modelling technique based on sweep operations was developed to obtain any part from a G-code file, without geometry limitations, in an automated way and with capabilities to obtain the 3D model in a few seconds.

One of the conclusions that can be extracted from the results is that the models obtained cannot be considered to be accurate representations of the printed parts, as they do not take into account dimensional variations. On the other hand, the models are not comparable, as they provide very different results depending on the methodology or type of mesh used. VOLCO modelling gives stiffer models due to the expansion of the material at the joints. However, the elastic modulus of the models follows the same pattern as the experimental one. In other words, even though they are not proportional, the most rigid model corresponds to the most rigid printed part in both methodologies. Therefore, it is possible to determine which will be the stiffest geometry.

Although geometry-based modelling is faster and more computationally efficient in simple geometries, it also presents more meshing problems than the voxel-based one. These meshing problems can be solved by reducing the seed size or the application of “virtual topology”; however, in certain cases, this does not work either.

Related to the dimensional accuracy, the volume differences between the models and the theoretical reference are negligible. In the CAD model, the volume is very close to the one expected in the G-code file. On the other hand, in the voxelised model, a manual adjustment of the volume through the spline function is required.

For all these reasons, if the objective is to find the fastest and easiest modelling technique to design and optimise a part to be printed with FDM technology, the new automatic CAD-based modelling is the preferred option. However, for small and complex models, voxel-based modelling is the most suitable option.

Following this research, the next steps need to be focused on optimising 3D printed parts before manufacturing, using the DECODE modelling technique (or VOLCO for small models) and FEA to drive the optimisation, thereby reducing the experimental work and improving the cost-efficiency of the process.

## Figures and Tables

**Table 1:** Material Properties of PCL.

Material Properties of PCL-Regemat 3D	
Molecular weight	50,000 g/mol
Density	1.1 g/cm^3^
Tensile strength	45 MPa
Elongation at yield	15%
Tensile modulus	350 MPa
IZOD impact strength (notched)	8 kJ/m^2^
Shore D hardness	46
Heat deflection temperature (0.45 MPa)	57 °C

**Table 2:** Printing Parameters of Scaffolds.

Print Settings		
**Common Settings to All the Parts**		
External perimeter extrusion width		0.44 mm (0.39 mm^3^/s)
Perimeter extrusion width		0.48 mm (0.88 mm^3^/s)
Infill extrusion width		0.48 mm (2.51 mm^3^/s)
Filament diameter		1.75 mm
Infill speed		20 mm/s
Nozzle diameter		0.4 mm
Temperature		190 °C
First layer height		0.35 mm
Fill angle		90°
**Particular Settings**		
50_rect	Fill density	50%
	Fill pattern	Rectilinear
60_rect	Fill density	40%
	Fill pattern	Rectilinear
50_gyr	Fill density	50%
	Fill pattern	Gyroid

**Table 3:** Printing Parameters of Test Specimen.

Print Settings	
External perimeter extrusion width	0.48 mm (1.87 mm^3^/s)
Perimeter extrusion width	0.48 mm (3.74 mm^3^/s)
Infill extrusion width	0.48 mm (3.74 mm^3^/s)
Filament diameter	1.75 mm
Infill speed	30 mm/s
Nozzle diameter	0.4 mm
Temperature	200 °C
Layer height	0.3 mm
First layer height	0.3 mm
Number of perimeters/solid layers	1
Fill angle	0°
Fill density	20%
Fill pattern	Concentric

**Table 4:** Comparison of Simulation Results.

Modelling Strategy	Reaction Force (N)
Whole-part modelling	13.4898
Line-by-line modelling	13.4721
Line-by-line modelling with corner revolutions	13.4759
Modelling by sections	13.4898

**Table 5:** Mesh Assignment.

Part	Modelling Technique	Mesh Type	Seed Size (mm)	Virtual Topology
50_rect	DECODE	C3D10	0.3	Yes
		C3D4	0.4	No
	VOLCO	C3D4	0.05	No
60_rect	DECODE	C3D10	0.1	No
		C3D4	0.1	No
	VOLCO	C3D4	0.05	No
50_gyr	DECODE	C3D10	-	-
		C3D4	0.05	No
	VOLCO	C3D4	0.05	No

**Table 6:** CPU Time and Memory Required to Model and Simulate the Scaffolds.

Part	Modelling Technique	Mesh Type	Coordinates Generation (s)	Modelling Process (s)	Simulation Process (s)	Total Time (h:mm:ss)	Minimum Memory Required (MB)
50_rect	DECODE	C3D10	10	55	255	0:05:10	1925
		C3D4			477	0:08:52	326
	VOLCO	C3D4		2339	4967	2:01:46	9549
60_rect	DECODE	C3D10	7	33	1559	0:26:32	7491
		C3D4			319	0:05:52	1238
	VOLCO	C3D4		959	2955	1:05:14	7985
50_gyr	DECODE	C3D10	7	338	-	-	-
		C3D4			2970	0:55:08	6905
	VOLCO	C3D4		920	2280	0:53:20	6901

**Table 7:** Number of voxels according to dimensions and set voxel size.

Type	Dimensions (mm)	Voxel Size (μm)	Min. No. Voxels (Memory Required) vs. Max. No. Elements (Memory Available)
Specimen	80 × 10 × 4	100	3.2 × 10^6^ (24.41 MB) < 5.86 × 10^9^ (44,713 MB)
Specimen	80 × 10 × 4	5	2.56 × 10^10^ (190.73 GB) > 5.86 × 10^9^ (43.66 GB)
Large part	200 × 250 × 500	100	2.5 × 10^10^ (186.26 GB) > 5.86 × 10^9^ (43.66 GB)
Corner part	181 × 181 × 181	100	5.93 × 10^10^ (44.18 GB) > 5.86 × 10^9^ (43.66 GB)

**Table 8:** Part Simulation Feasibility.

Part	VOLCO				DECODE			
	STL Generation	Abaqus/CAE 6.14-1			PY Generation	Abaqus/CAE 6.14-1		
		Importation	Meshing	FEA		Importation	Meshing	FEA
Specimen	✓	✓	✓	✓	✓	✓	✓	✓
Large part					✓			
Corner part					✓	✓		
Rectilinear scaffolds	✓	✓	✓	✓	✓	✓	✓	✓
Gyroid scaffold	✓	✓	✓	✓	✓	✓		
Mini specimen	✓	✓	✓	✓	✓	✓	✓	✓

**Table 9:** Comparison of Volumes.

Part	Theoretical Volume (mm^3^)	Experimental Volume (mm^3^)	Modelling Technique	Model Volume (mm^3^)	Theoretical Deviation	Experimental Deviation
	Vt	Ve		Vm	|Vm − Vt|/Vt × 100	|Vm − Ve|/Ve × 100
50_rect	278.8	274.91	DECODE	278.46	0.12%	1.29%
			VOLCO	278.8	0%	1.42%
60_rect	231.16	224.18	DECODE	230.95	0.09%	3.02%
			VOLCO	231.15	0%	3.11%
50_gyr	188.83	183.64	DECODE	188.81	0%	2.82%
			VOLCO	188.83	0%	2.83%

**Table 10:** Comparison of Dimensions.

Part	Dimension	Theoretical (mm)	Real (mm)	Modelling Technique	Model (mm)	Theoretical Deviation	Real Deviation
		T	R		M	|M − T|/T × 100	|M − R|/R × 100
50_rect	Diameter	10	7.06	DECODE	10.104	1.04%	43.12%
				VOLCO	10.025	0.25%	42.00%
	Height	7	6.04	DECODE	6.95	0.71%	15.07%
				VOLCO	6.95	0.71%	15.07%
60_rect	Diameter	10	6.83	DECODE	10.098	0.98%	47.85%
				VOLCO	10.05	0.50%	47.14%
	Height	7	5.91	DECODE	6.95	0.71%	17.60%
				VOLCO	6.95	0.71%	17.60%
50_gyr	Diameter	10	7.42	DECODE	10.832	8.32%	45.98%
				VOLCO	10.075	0.75%	35.78%
	Height	7	5.63	DECODE	6.954	0.66%	23.52%
				VOLCO	6.95	0.71%	23.45%

**Table 11:** Comparison of Elastic Modulus.

Part	Experimental Young Modulus (MPa)	Modelling Technique	Mesh Type	Model Young Modulus (MPa)	Deviation
	Er			Em	|Em − Er|/Er × 100
50_rect	58.88	DECODE	C3D10	49.16	16.52%
			C3D4	85.19	44.68%
		VOLCO	C3D4	103.4	75.61%
60_rect	55.58	DECODE	C3D10	28.93	47.95%
			C3D4	40.56	27.03%
		VOLCO	C3D4	71.09	27.91%
50_gyr	39.21	DECODE	C3D10	-	-
			C3D4	11.88	69.70%
		VOLCO	C3D4	41.71	6.37%

## Data Availability

The data presented in this study are available within the article.

## Supplementary Materials

The following are available online at https://www.mdpi.com/article/10.3390/ma14195670/s1: Figure S1. FEA result of 50_gyr scaffold (base reaction forces and deformation) modelled by DECODE with C3D4 mesh type; Figure S2. FEA result of 50_gyr scaffold (base reaction forces and deformation) modelled by VOLCO with C3D4 mesh type; Figure S3. FEA result of 50_rect scaffold (base reaction forces and deformation) modelled by DECODE with C3D4 mesh type; Figure S4. FEA result of 50_rect scaffold (base reaction forces and deformation) modelled by DECODE with C3D10 mesh type; Figure S5. FEA result of 50_rect scaffold (base reaction forces and deformation) modelled by VOLCO with C3D4 mesh type; Figure S6. FEA result of 60_rect scaffold (base reaction forces and deformation) modelled by DECODE with C3D4 mesh type; Figure S7. FEA result of 60_rect scaffold (base reaction forces and deformation) modelled by DECODE with C3D10 mesh type; Figure S8. FEA result of 60_rect scaffold (base reaction forces and deformation) modelled by VOLCO with C3D4 mesh type; Figure S9. FEA result (stress and deformation) of test specimen; Figure S10. Specimen modelling and FEA process by VOLCO; Figure S11. Specimen modelling and FEA process by DECODE; Figure S12. G-code generation of large part; Figure S13. Corner part modelling process.
