# Supplementary figures and images for: Comparison of CAD and Voxel-Based Modelling Methodologies for the Mechanical Simulation of Extrusion-Based 3D Printed Scaffolds
Source: Materials (Basel). 2021 Sep 29;14(19):5670. doi: 10.3390/ma14195670 (PMC8510365; doi:10.3390/ma14195670)

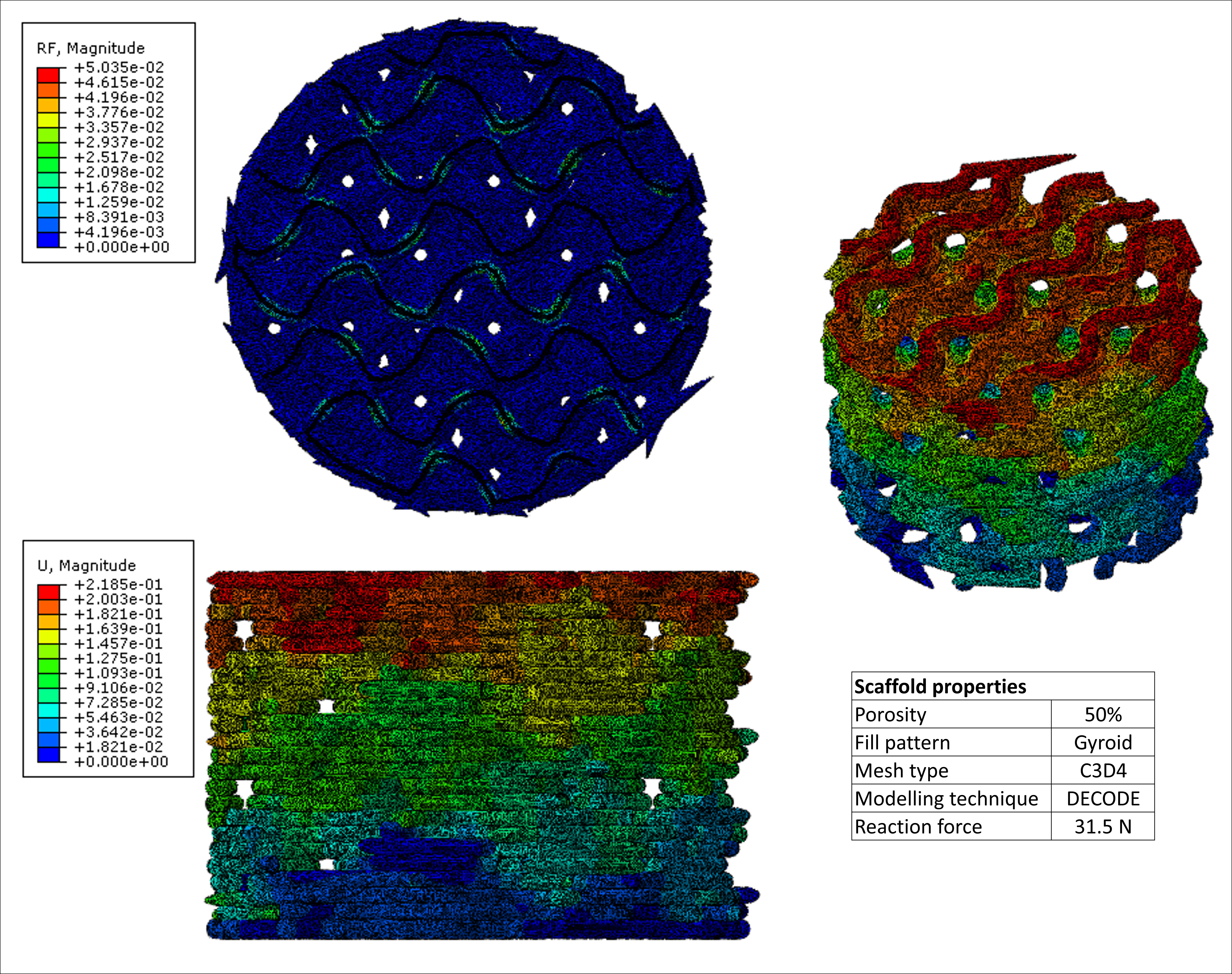

Supplement: Supplementary file 1 [file materials-14-05670-s001.zip › FIG S1.tif]

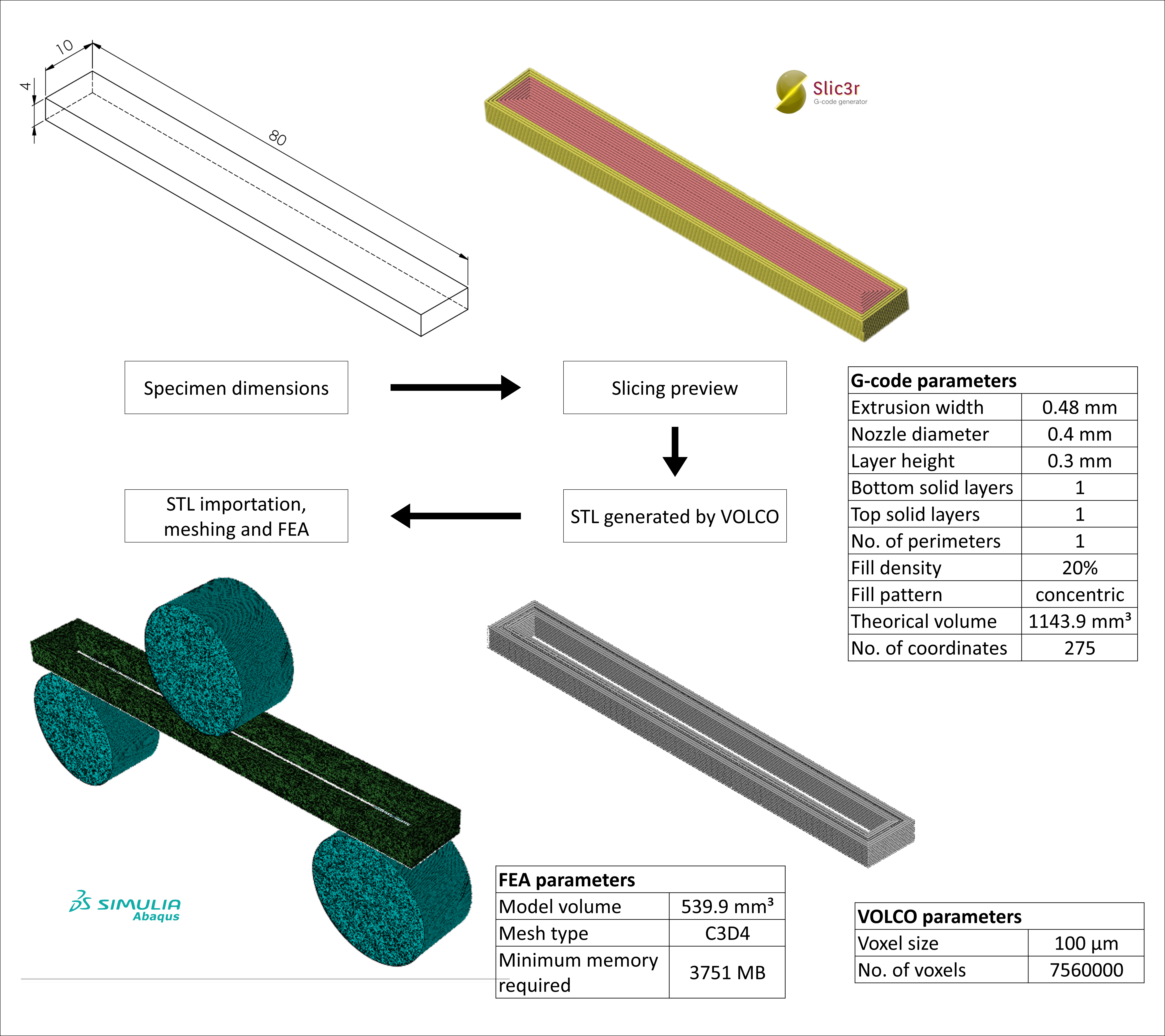

Supplement: Supplementary file 1 [file materials-14-05670-s001.zip › FIG S10.tif]

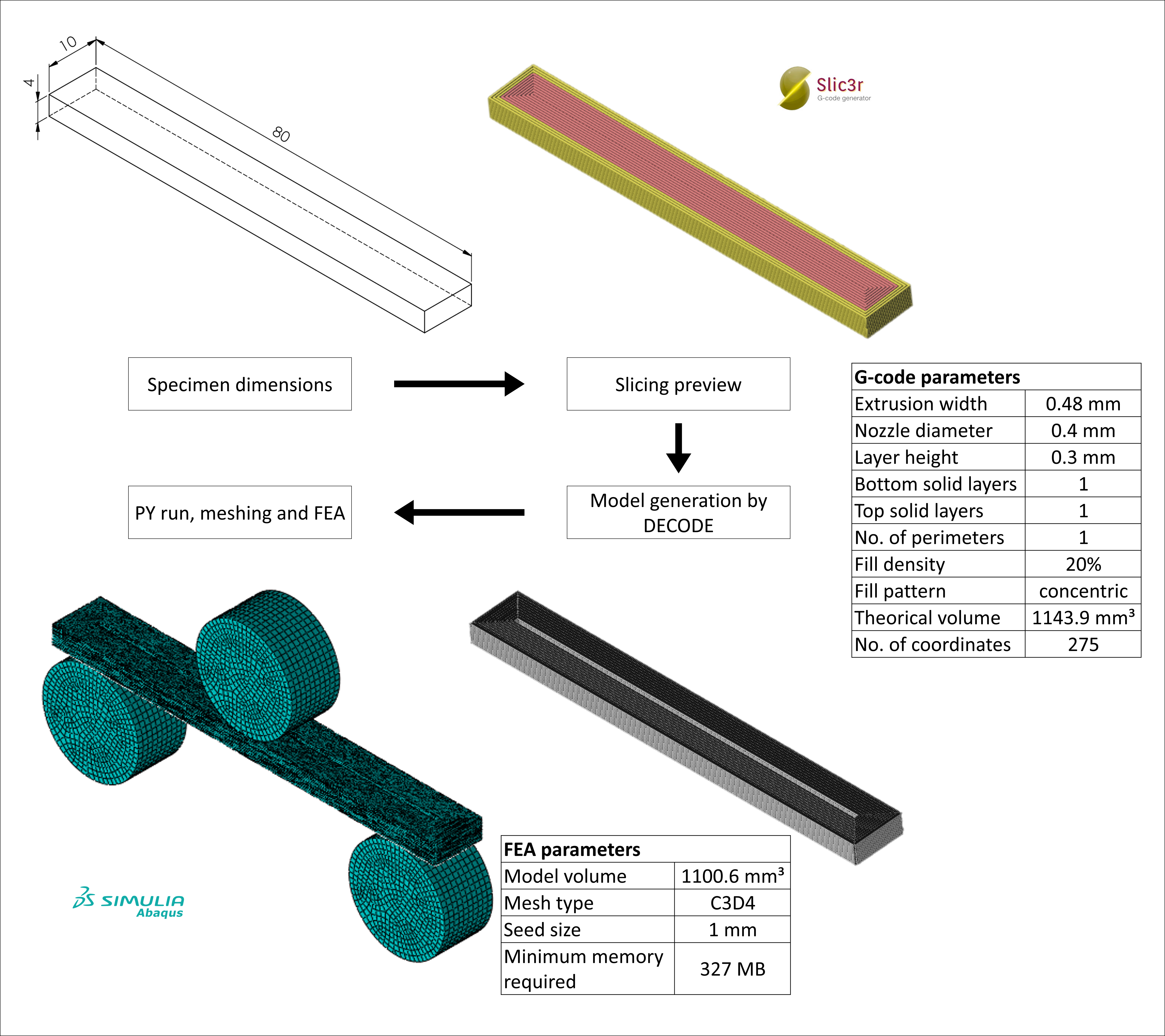

Supplement: Supplementary file 1 [file materials-14-05670-s001.zip › FIG S11.tif]

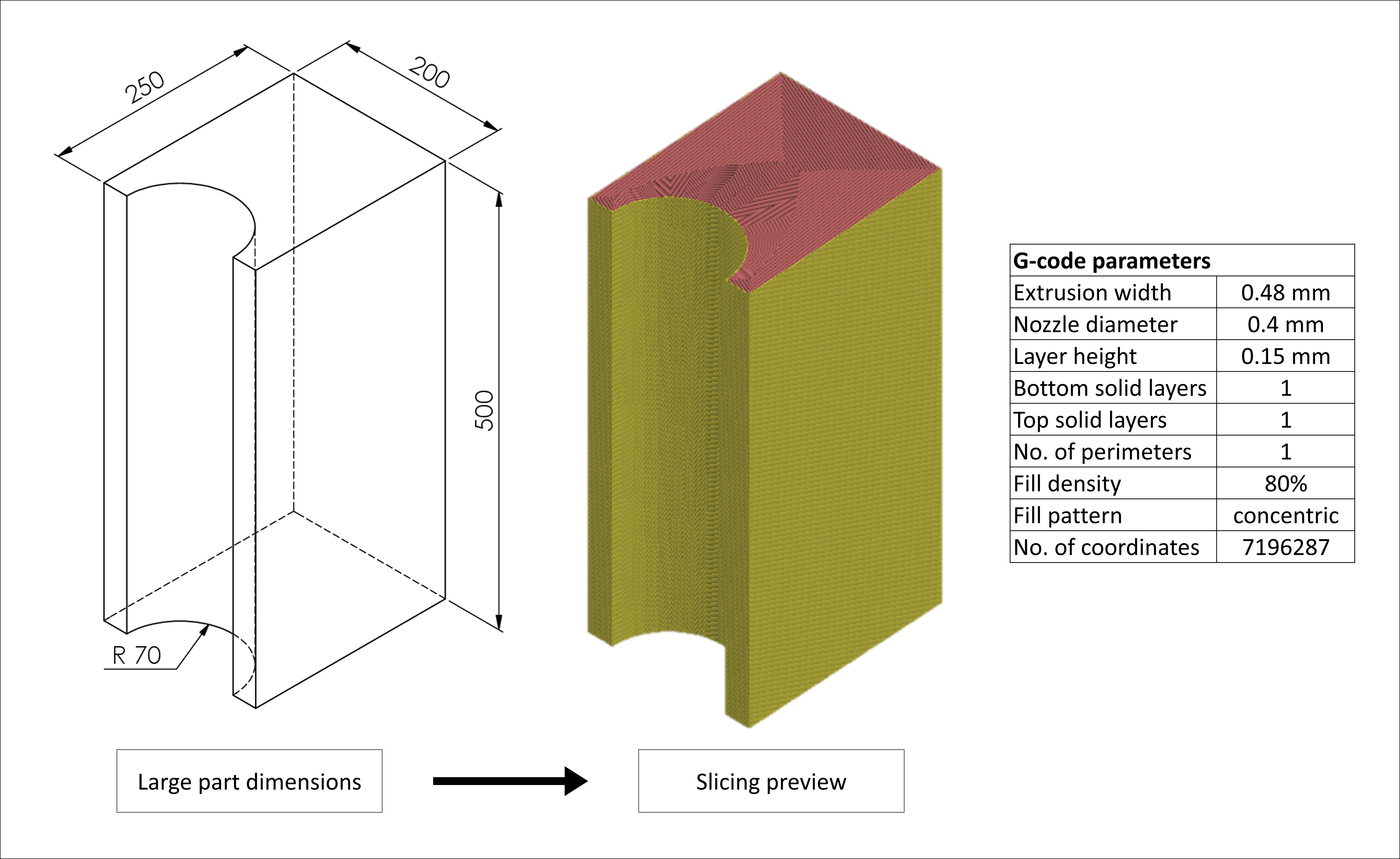

Supplement: Supplementary file 1 [file materials-14-05670-s001.zip › FIG S12.tif]

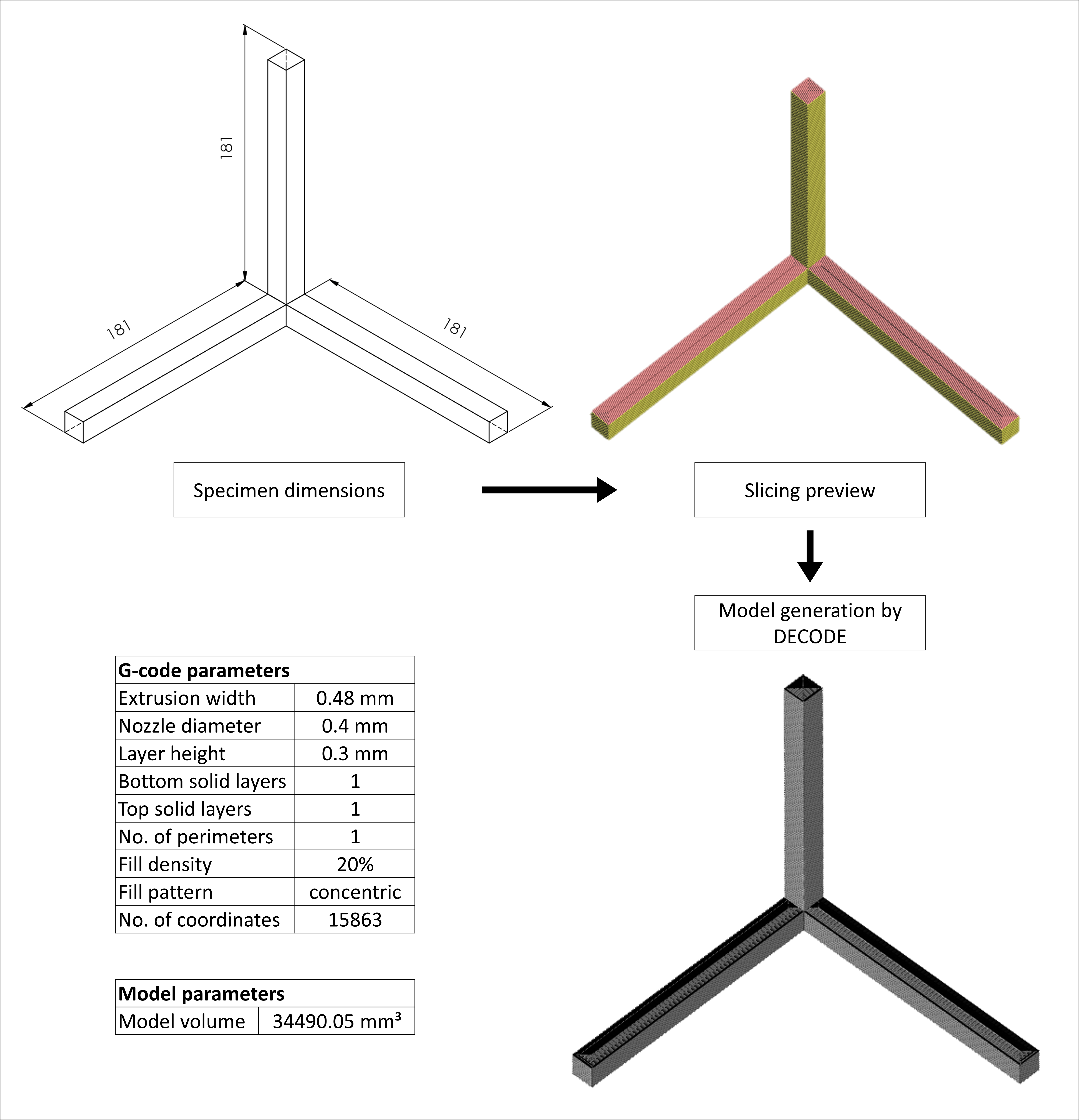

Supplement: Supplementary file 1 [file materials-14-05670-s001.zip › FIG S13.tif]

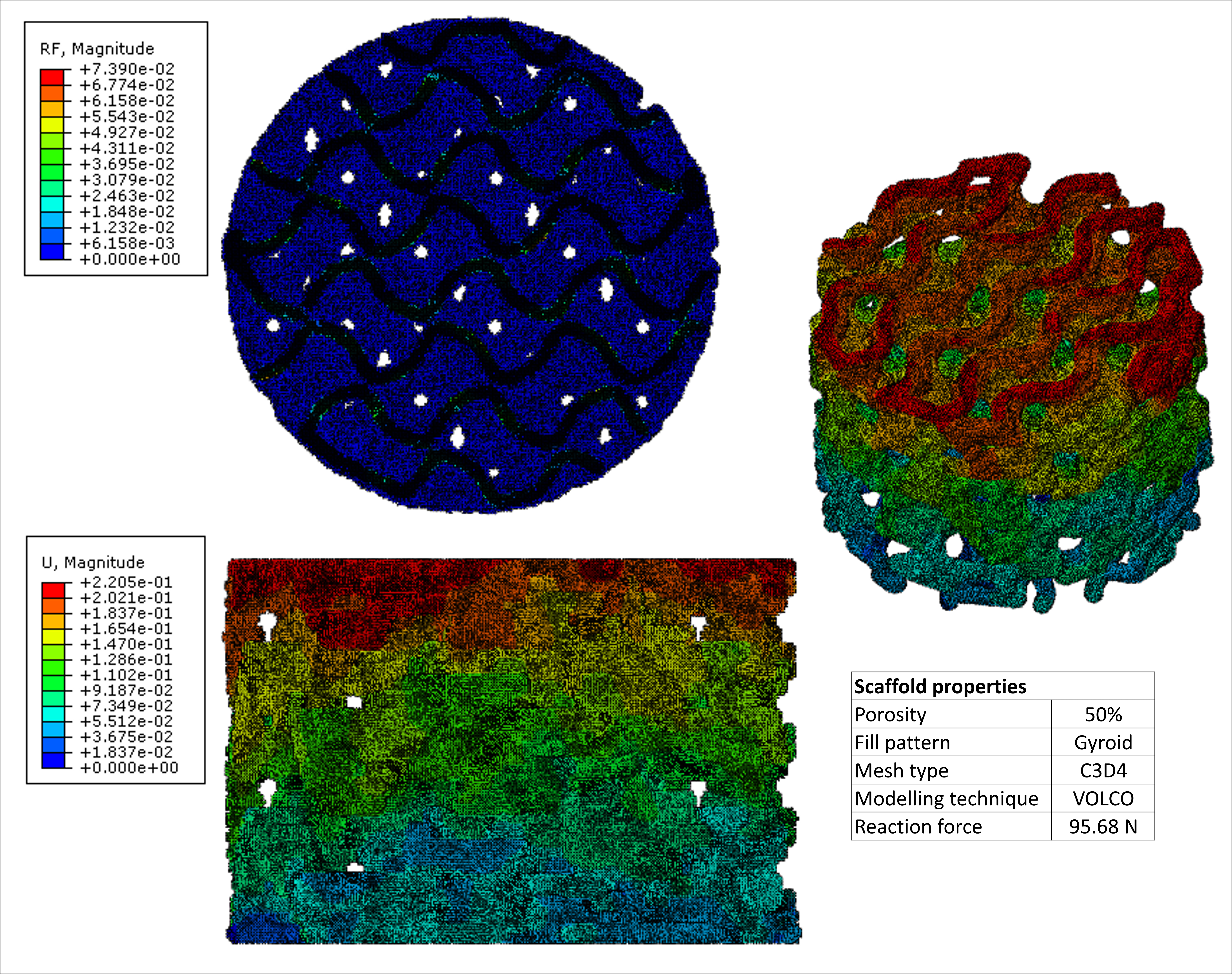

Supplement: Supplementary file 1 [file materials-14-05670-s001.zip › FIG S2.tif]

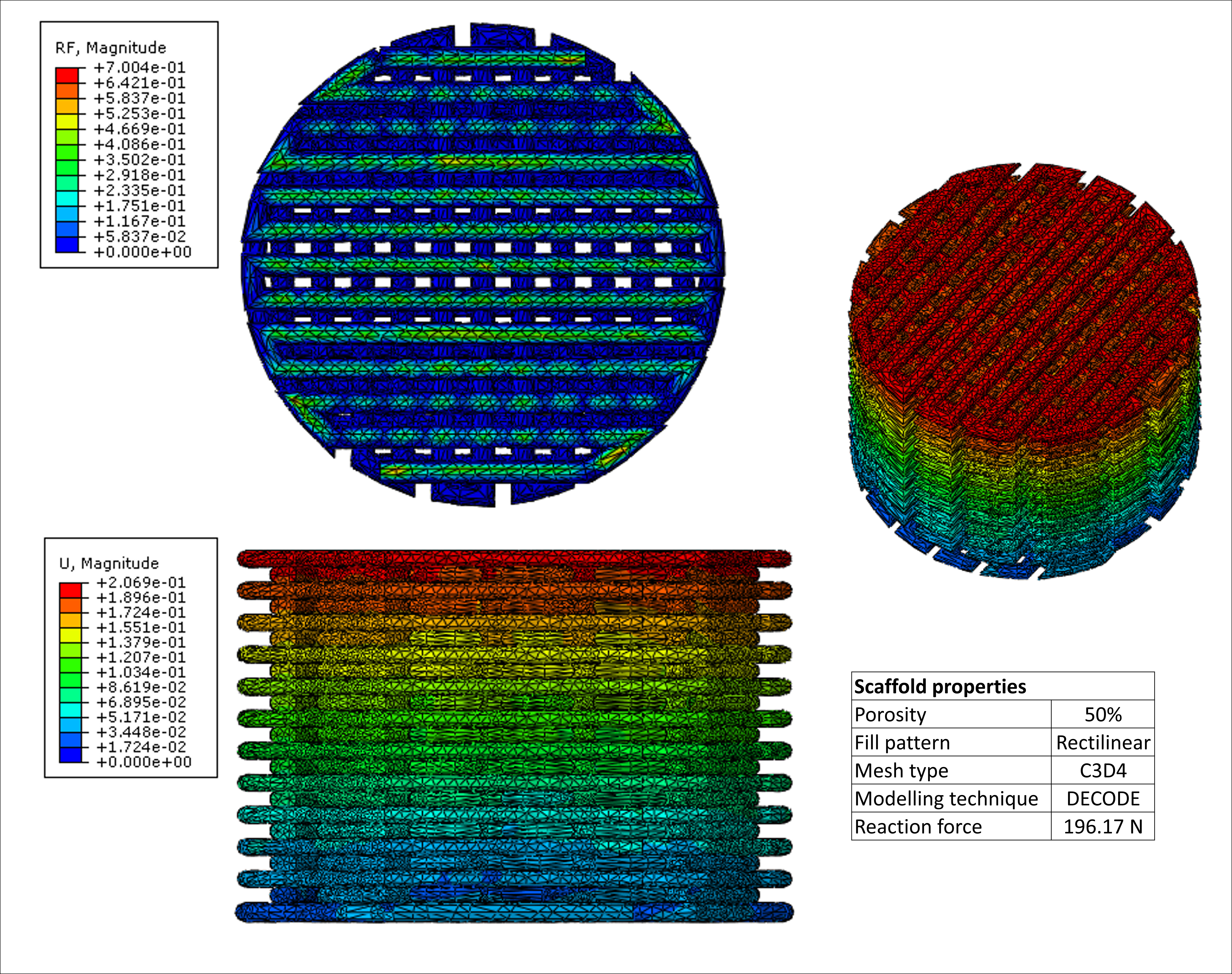

Supplement: Supplementary file 1 [file materials-14-05670-s001.zip › FIG S3.tif]

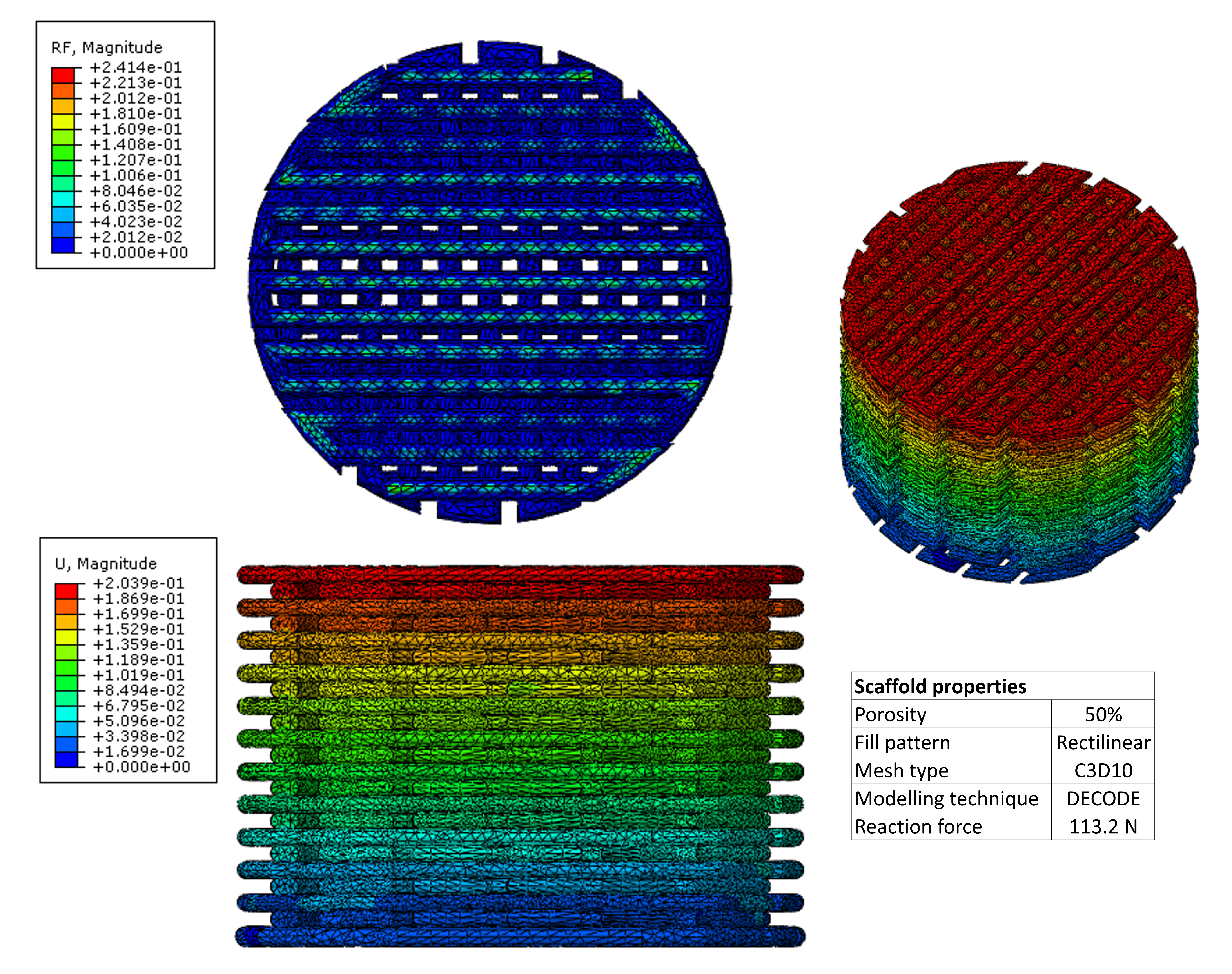

Supplement: Supplementary file 1 [file materials-14-05670-s001.zip › FIG S4.tif]

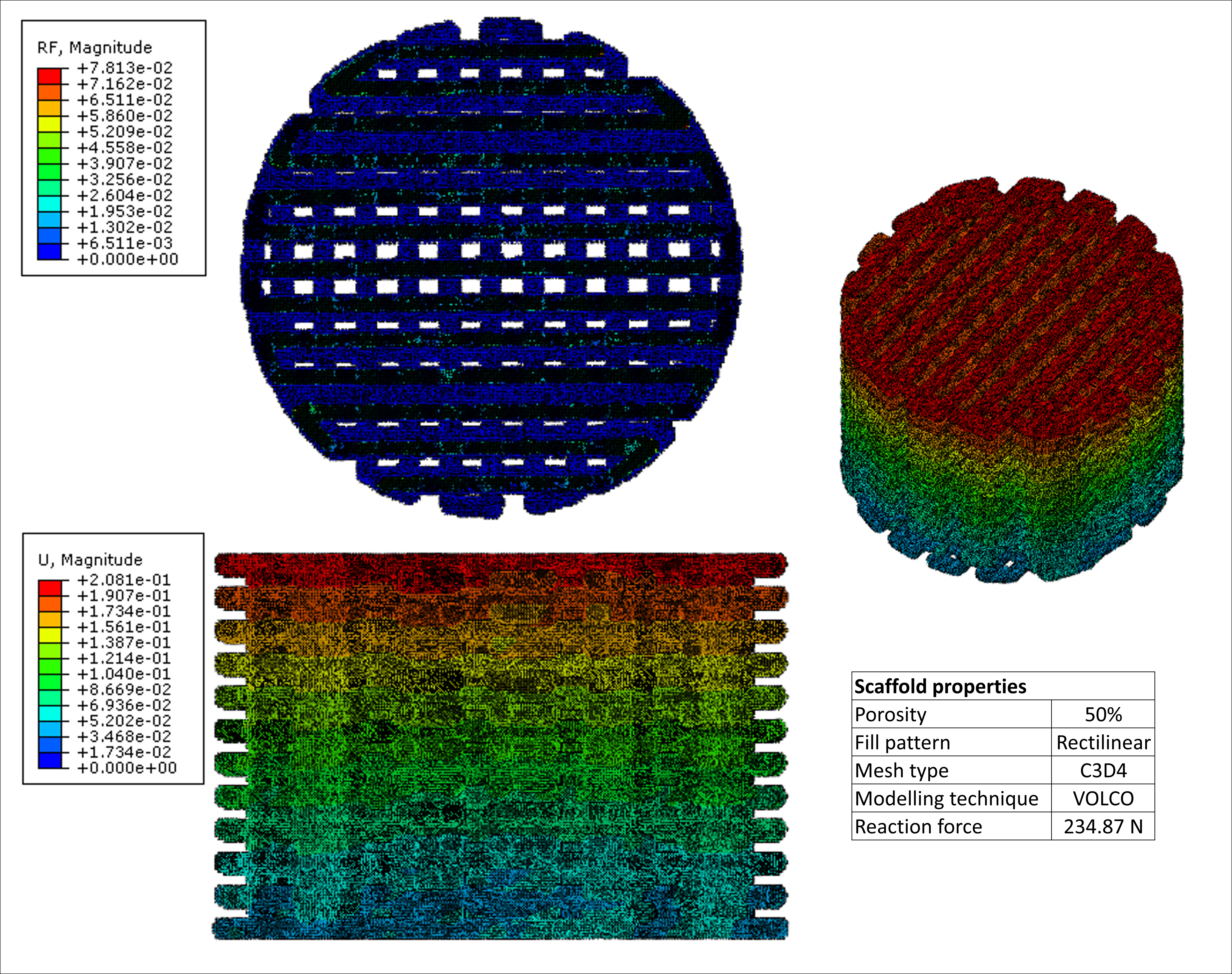

Supplement: Supplementary file 1 [file materials-14-05670-s001.zip › FIG S5.tif]

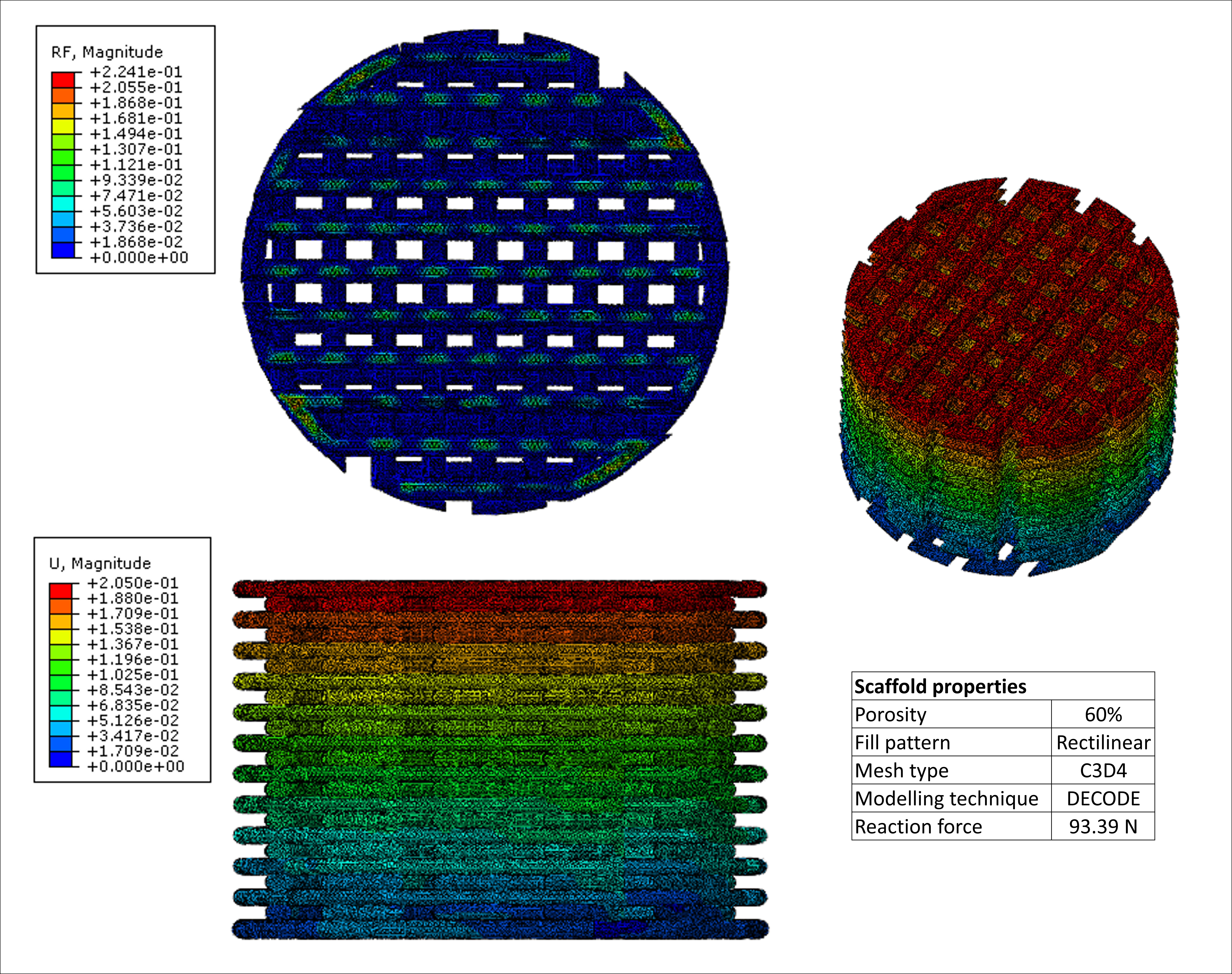

Supplement: Supplementary file 1 [file materials-14-05670-s001.zip › FIG S6.tif]

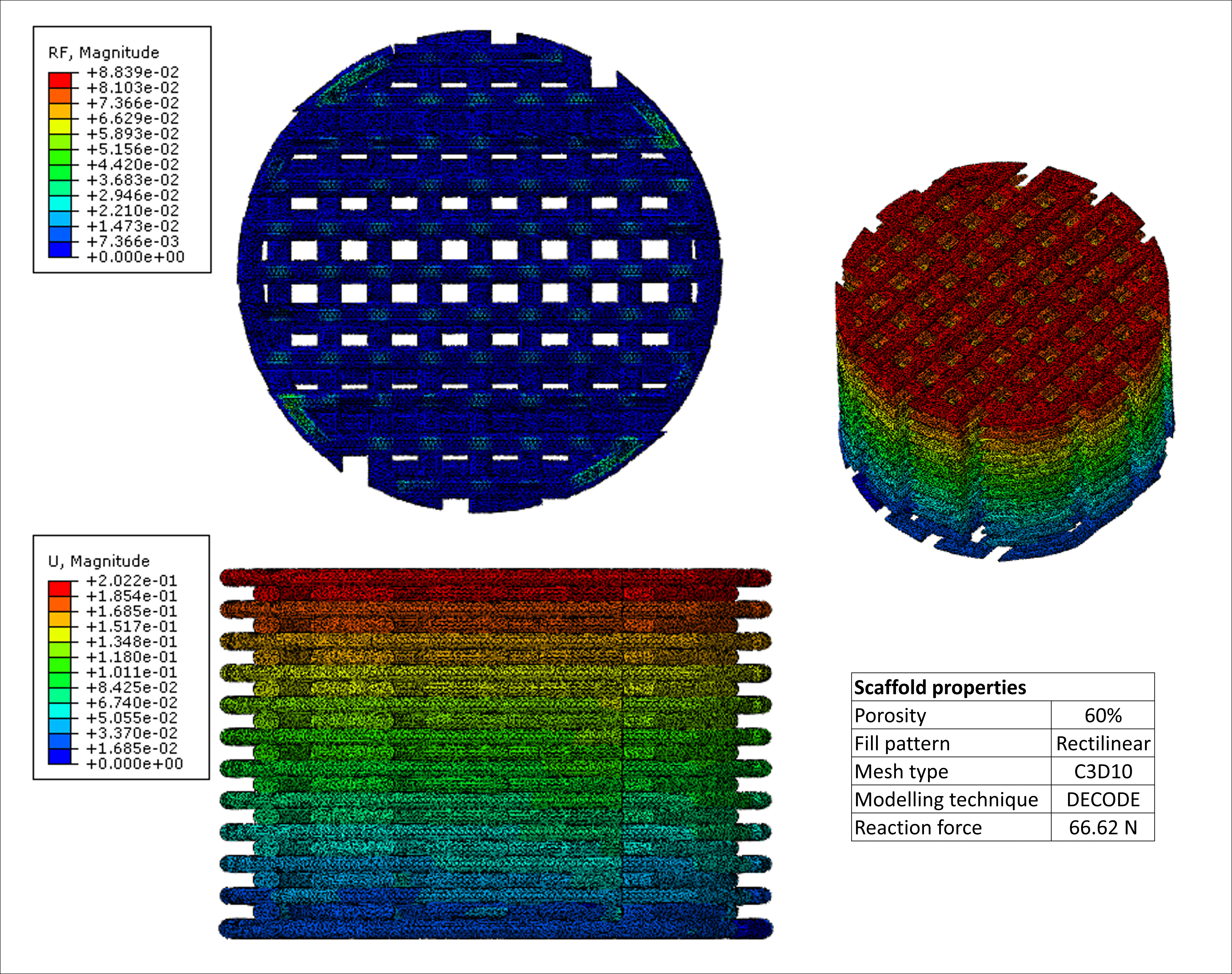

Supplement: Supplementary file 1 [file materials-14-05670-s001.zip › FIG S7.tif]

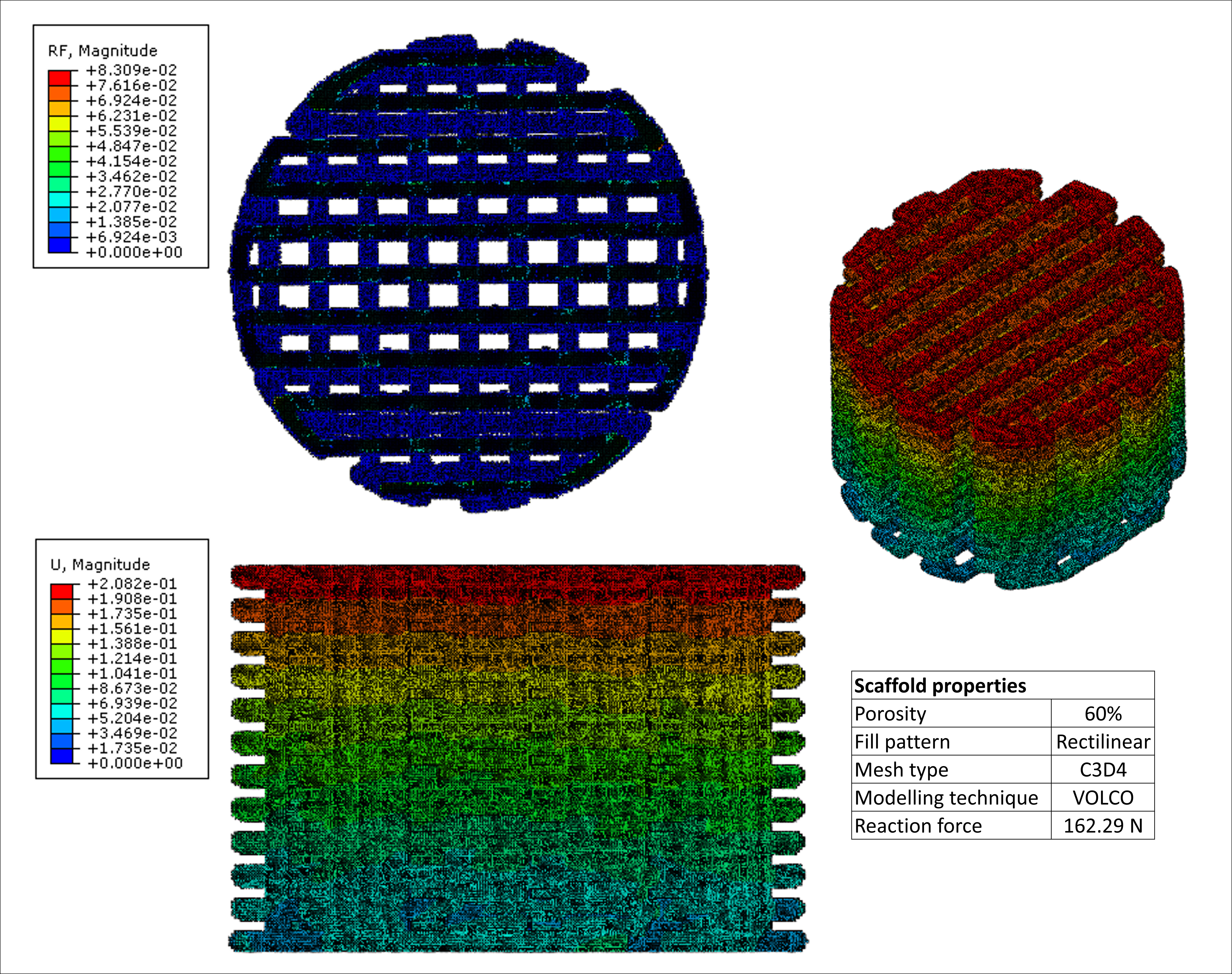

Supplement: Supplementary file 1 [file materials-14-05670-s001.zip › FIG S8.tif]

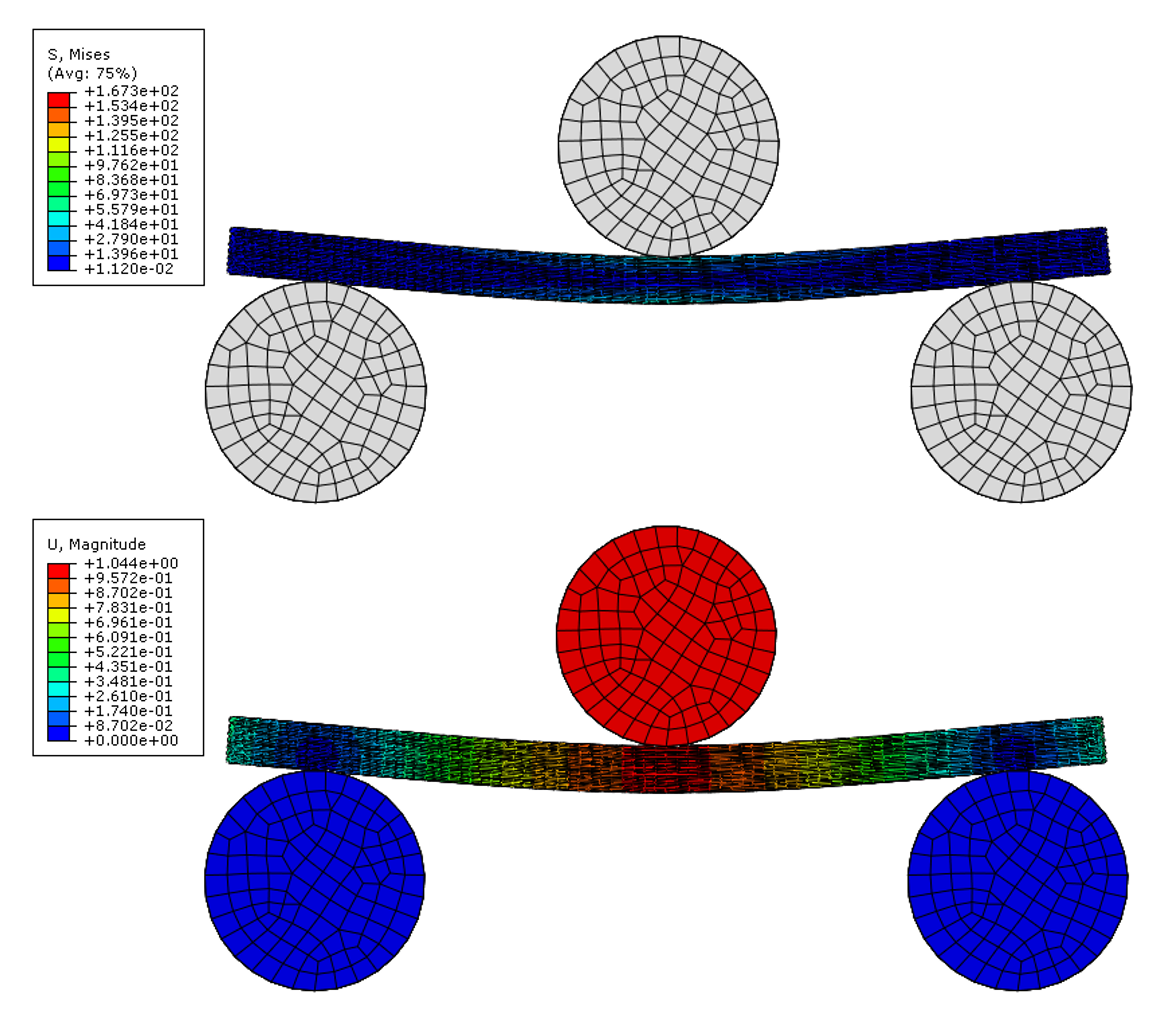

Supplement: Supplementary file 1 [file materials-14-05670-s001.zip › FIG S9.tif]
